# Universal Poisson statistics of a passive tracer diffusing in dilute active suspensions

**DOI:** 10.1073/pnas.2308226120

**Published:** 2023-12-04

**Authors:** Adrian Baule

**Affiliations:** ^a^School of Mathematical Sciences, Queen Mary University of London, London E1 4NS, United Kingdom

**Keywords:** statistical mechanics, stochastic processes, active matter, nonequilibrium

## Abstract

The statistics of a passive tracer immersed in a suspension of active particles (swimmers) is derived from first principles by considering a perturbative expansion of the tracer interaction with the microscopic swimmer field. To first order in the swimmer density, the tracer statistics is shown to be exactly represented by a spatial Poisson process combined with independent tracer—swimmer scattering events, rigorously reducing the multiparticle dynamics to two-body interactions. The Poisson representation is valid in any dimension, for arbitrary interaction forces and for a large class of swimmer dynamics. The framework not only allows for the systematic calculation of the tracer statistics in various dynamical regimes but highlights in particular surprising universal features that are independent of the swimmer dynamics such as a time-independent velocity distribution.

A small tracer immersed in a suspension of swimming microorganisms such as algae or bacteria exhibits a diffusion process that is strikingly different from Brownian motion, manifest in a displacement distribution with strong non-Gaussian features (1–3). While a range of theoretical approaches have been applied to calculate the effective diffusivity of the tracer (4, 5), determining its detailed statistics from first principles for realistic forces and swimmer dynamics has been challenging. Previous work has described the tracer motion by a generalized Langevin equation, which is related to an assumption of time-scale separation and has been derived, e.g., for inertial dynamics and a linear coupling between the tracer and swimmers (6, 7), and for overdamped dynamics and more general forces in one dimension (8). The non-Gaussian features have been explained based on rare and nonlinear scattering events whose superposition is not subject to the central limit theorem (9). However, a quantitative treatment of the multiparticle scattering dynamics relies on a phenomenological assumption of independent scatterings and simplified swimmer dynamics such as swimmers moving in straight lines (9–11). Here, I show that under very general assumptions, a universal Poisson representation of the tracer dynamics can be derived when the swimmer density is low.

The setup consists of N active particles (swimmers) and a passive tracer in d dimensions suspended in a viscous fluid inside a box of length L and volume $V=L^{d}$. In line with previous approaches (9–11), I assume: a) A dilute suspension of swimmers with small number density $\overset{¯}{\rho }=N/V$, such that mutual interactions between the swimmers do not play a role. b) A passive tracer that does not interact with the swimmer dynamics. c) Weak thermal noise that can be neglected compared with the active and viscous forces. The overdamped equation of motion for the tracer position X(t) thus takes the form:

[1] $$\begin{array}{c} \dot{X}\left(t\right)=\mu \sum_{i=1}^{N}F\left(Y_{i}\left(t\right)-X\left(t\right),{\dot{Y}}_{i}\left(t\right)\right), \end{array}$$

where all quantities have been made dimensionless by suitable rescaling; see *SI Appendix*, 1. In Eq. **1**, μ denotes the tracer’s mobility coefficient and F the force between the tracer and the ith swimmer at position $Y_{i}$, which can include short-range, long-range, attractive, and hydrodynamic interactions. The swimmer dynamics $Y_{i}\left(t\right)$ is assumed to follow a stochastic process and can be identified, e.g., with the widely studied models of active Brownian particles (ABPs), run-and-tumble particles (RTPs), and active Ornstein–Uhlenbeck particles (AOUPs), or any other Markovian or non-Markovian dynamics, e.g., due to colored noise, provided $Y_{i}\left(t\right)$ satisfies translation invariance and is not generated due to interactions with other swimmers. The initial conditions are X(0)=0, positions $Y_{i}\left(0\right)$ distributed uniformly in the volume, and velocities ${\dot{Y}}_{i}\left(0\right)$ drawn from a distribution p(v).

To ease the notation, I focus on forces without ${\dot{Y}}_{i}$ dependence and denote the time-dependence by a subscript ($X\left(t\right)\equiv X_{t}$). Due to the generality of forces and swimmer dynamics considered, Eq. **1** describes a large variety of scenarios, such as swimming microorganisms or self-propelled colloids interacting with nutrients, passive cargo, or degraded plastic. Dilute conditions have indeed been realized in the experiments (1–3) and others (*SI Appendix*, 1), which can be readily used to test some of the predictions derived below.

The central quantity is the characteristic functional (CF)

[2] $$\begin{array}{c} \psi_{\dot{X}}\left[k\right]=\langle \exp\left\{i\int_{0}^{t}duk_{u}\cdot {\dot{X}}_{u}\right\}\rangle , \end{array}$$

which can be used to determine, e.g., the characteristic functions of the tracer velocity ${\dot{X}}_{\tau }$ and displacements $\Delta X_{\tau }=X_{\tau }-X_{0}$, by setting $k_{u}=\delta \left(\tau -u\right)k$ and $k_{u}=\Theta \left(\tau -u\right)k$, respectively. An exact expression for the CF that is valid under the assumptions above is derived as follows:

1. The exact microscopic density field of the swimmers $\rho_{t}\left(x\right)=\sum_{i=1}^{N}\delta \left(x-Y_{i}\left(t\right)\right)$ is introduced, so that Eq. **1** can be likewise written as ${\dot{X}}_{t}=\mu \int dxF\left(x-X_{t}\right)\rho_{t}\left(x\right)$. Using the Martin–Siggia–Rose–Janssen–De Dominicis (MSRJD) formalism the average with respect to the stochastic trajectories of the tracer and swimmers is expressed as a field theory, which implicitly requires to take the thermodynamic limit V→∞,N→∞, while keeping the swimmer density $\overset{¯}{\rho }=$ const. Averages of observables A of the tracer dynamics are then formally expressed as (12)
  [3] $$\begin{array}{c} \langle A[X]\rangle =\langle {\langle A\left[X\right]e^{i\mu \int_{0}^{t}ds\int dxg_{s}\cdot F\left(x-X_{s}\right)\rho_{s}\left(x\right)}\rangle }_{0}\rangle , \end{array}$$
  where the notation ${\left\langle ...\right\rangle }_{0}$ is used for the average $\int D\left[\frac{g}{2\pi }\right]\int D\left[X\right]...\delta \left(X\left(0\right)\right)e^{-i\int_{0}^{t}dsg_{s}\cdot {\dot{X}}_{s}}$ and $g_{s}$ denotes the conjugate field introduced in the MSRJD formalism to constrain trajectories X(s) to the equation of motion of the tracer (12). The remaining average ⟨...⟩ in Eq. **3** is taken with respect to the swimmer dynamics incorporated in $\rho_{t}\left(x\right)$.
2. The CF is obtained by applying Eq. **3** to the observable $A\left[X\right]=e^{i\int_{0}^{t}duk_{u}\cdot {\dot{X}}_{u}}$. Expanding then the exponential of Eq. **3** yields a perturbation series that contains at the nth order the n-point correlation function of $\rho_{t}\left(x\right)$. To first order in $\overset{¯}{\rho }$, these n-point functions are given as (*SI Appendix*, 2)
  [4] $$\begin{array}{cc} & \langle \rho_{t_{1}}\left(x_{1}\right)\cdots \rho_{t_{n}}\left(x_{n}\right)\rangle \\ & =\overset{¯}{\rho }\int dy{\langle \delta \left(x_{1}-Y_{t_{1}}\right)\cdots \delta \left(x_{n}-Y_{t_{n}}\right)\rangle }_{Y_{0}=y}. \end{array}$$
3. Remarkably, after evaluating explicitly the averages ${\left\langle ...\right\rangle }_{0}$ in each term of the perturbative expansion, the series can be resummed leading to the exact result (*SI Appendix*, 3)
  [5] $$\begin{array}{c} \ln\psi_{\dot{X}}\left[k\right]=\overset{¯}{\rho }\int dy(\langle e^{i\int_{0}^{t}duk_{u}\cdot f^{*}\left[Y_{u}\right]}\rangle_{Y_{0}=y}-1). \end{array}$$
  In Eq. **5**, $f^{*}$ is defined as $f^{*}\left[Y_{t}\right]=\frac{d}{dt}X^{*}\left(t\right)$, where $X^{*}$ is the formal solution of the two-body problem
  [6] $$\begin{array}{c} {\dot{X}}_{t}=\mu F\left(Y_{t}-X_{t}\right), \end{array}$$
  describing the motion of the tracer interacting with a single swimmer moving along the trajectory $Y_{t}$ starting at $Y_{0}=y$. Eq. **5** is exact up to first order in $\overset{¯}{\rho }$ and implies that the tracer dynamics can be expressed as a stochastic equation in the form ${\dot{X}}_{t}=\sum_{y\in \Phi }f^{*}\left[Y_{t}\right]$, where Φ represents a spatial Poisson process with intensity $\overset{¯}{\rho }$, see *SI Appendix*, 4. The tracer thus moves due to independent scatterings with individual swimmers, which are distributed uniformly and independently with density $\overset{¯}{\rho }$. The simplification of the multiparticle dynamics to independent two-body interactions is not an assumption of the theory as, e.g., in the kinetic theory of gases, but an exact result of the calculation, and relies on the fact that the n-point functions reduce exactly to single-particle self-correlations to lowest order in $\overset{¯}{\rho }$ as expressed in Eq. **4**. When F includes a dependence on the swimmer velocity, Eq. **5** is valid in the same form, see *SI Appendix*, 5.

While evaluating the conditional average in Eq. **5** in general is not feasible for realistic interaction forces, well-controlled approximations are obtained, e.g., by solving the two-body problem Eq. **6** by Picard iteration (*SI Appendix*, Eq. **S18**) and expressing the solution as a Taylor series in μ leading to:

[7] $$f^{*}[Y_{t}]\;\;\approx \mu F(Y_{t})$$

[8] $$f^{*}[Y_{t}]\;\;\approx \mu F(Y_{t})-\mu^{2}\int_{0}^{t}du\nabla F{\left(Y_{t}\right)}^{\text{T}}F(Y_{u}),\;$$

where ∇F denotes the matrix ${\left(\right)}_{\mathrm{ij}}$ with entries $\partial_{i}F_{j}$. Such an expansion is physically relevant when the typical tracer velocity is much smaller than that of the swimmers, which is indeed realized for tracers of a few μm size interacting with microorganisms or active colloids in suspension, see *SI Appendix*, 1. The following predictions are derived:

(a) Existing literature results (9, 11) are obtained as special cases; see *SI Appendix*, 6. For straight-line swimmer motion $Y_{t}=y+v_{A}\hat{n}t$, where $v_{A}$ denotes the swim speed and $\hat{n}$ the swimmer orientation, and a hydrodynamic dipole force:
  [9] $$\begin{array}{c} F_{\mathrm{hyd}}\left(x,\hat{n}\right)=\frac{p}{x^{2}}\left(3\frac{{\left(\hat{n}\cdot x\right)}^{2}}{x^{2}}-1\right)\frac{x}{\left|x\right|}, \end{array}$$
  the colored Poisson process of ref. (11) is exactly recovered.
(b) In the adiabatic regime Eq. **7** the tracer velocity ${\dot{X}}_{\tau }$ exhibits universal time-independent statistics, see *SI Appendix*, 7. Setting $k_{u}=\delta \left(\tau -u\right)k$ in Eqs. **5** and **7** and using translation invariance of $Y_{t}$ yields the characteristic function
  [10] $$\begin{array}{c} \psi_{\dot{X}}\left(k\right)=\exp\left\{\overset{¯}{\rho }\int dy\left(e^{i\mu k\cdot F(y)}-1\right)\right\}, \end{array}$$
  which is valid for any F(x), in any dimension, and for any $Y_{t}$ as above. Eq. **10** is verified in 2d simulations for the Coulomb-like force $F_{\mathrm{cb}}\left(x\right)=\sigma \frac{x}{{\left|x\right|}^{3}}$ and $Y_{t}$ given as ABPs, RTPs, AOUPs, and correlated angle particles (CAPs). For CAPs, the orientation angle follows an Ornstein–Uhlenbeck process: ${\dot{Y}}_{t}=v_{A}\hat{n}\left(\theta_{t}\right)$, $\tau_{c}{\dot{\theta }}_{t}=-\theta_{t}+\sqrt{D_{\theta }}\xi_{t}$, where $\tau_{c}$ and $D_{\theta }$ denote the persistence time and diffusivity, respectively, and $\xi_{t}$ is Gaussian white noise. Evaluating Eq. **10** for $F_{\mathrm{cb}}$ yields the PDF of the velocity projections $v_{x}={\dot{X}}_{\tau }\cdot {\hat{e}}_{x}$
  [11] $$\begin{array}{c} P\left(v_{x}\right)=\frac{1}{2\pi }\int dke^{-ikv_{x}}\psi_{\dot{X}}\left(k{\hat{e}}_{x}\right)=\frac{1}{\pi }\frac{c\overset{¯}{\rho }\mu }{{\left(c\overset{¯}{\rho }\mu \right)}^{2}+v_{x}^{2}}, \end{array}$$
  with c=πσ, confirmed in simulations for all four swimmer models and different τ; see Fig. 1*A*.

**Fig. 1.**
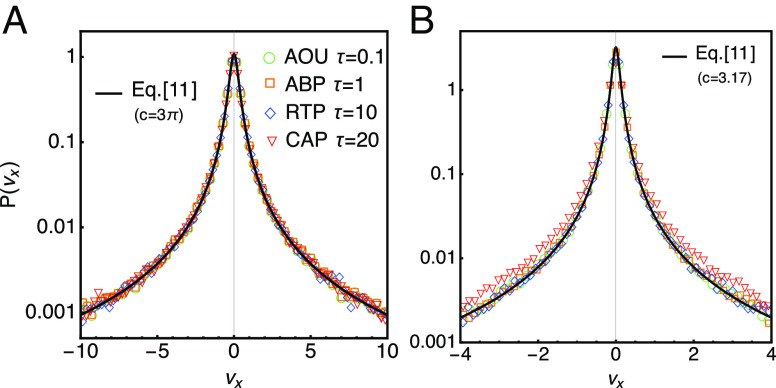
The PDF of the tracer velocities projected on the x-axis ($v_{x}={\dot{X}}_{\tau }\cdot {\hat{e}}_{x}$) measured in 2d simulations of Eq. **1** for two different F and four swimmer processes $Y_{i}\left(t\right)$ following ABPs, RTPs, AOUPs, and CAPs. (*A*) The Coulomb-like force $F_{\mathrm{cb}}=\sigma \frac{x}{{\left|x\right|}^{3}}$ leading to $P(v_{x})$ of Eqs. **11** and **10** with c=πσ, σ=3. (*B*) The hydrodynamic force $F_{\mathrm{hyd}}$ of Eq. **9** leading to $P(v_{x})$ of Eqs. **11** and **13** with c=3.17|p|, p=−1. For CAPs, the deviation from Eq. **11** can be tuned by changing the persistence time $\tau_{c}$. Symbols as in (*A*). See *SI Appendix*, 7 for details.

When F depends on the swimmer velocity, Eq. **7** is $f^{*}\left[Y_{t}\right]\approx \mu F\left(Y_{t},{\dot{Y}}_{t}\right)$ and Eq. **5** becomes

[12] $$\begin{array}{cc} & \ln\psi_{\dot{X}}\left(k\right)\\ & \;=\overset{¯}{\rho }\int dy\left(\int dv^{\prime }\int dvG_{v}\left(v^{\prime },\tau |v\right)p\left(v\right)e^{i\mu k\cdot F(y,v^{\prime })}-1\right), \end{array}$$

where $G_{v}$ is the propagator of ${\dot{Y}}_{t}$. Eq. **12** is not in general time-independent. However, for a class of $Y_{t}$ processes in 2d and for forces that depend only on $\hat{n}$ like Eq. **9**, Eq. **12** becomes again independent of the details of $Y_{t}$ and τ

[13] $$\begin{array}{c} \ln\psi_{\dot{X}}\left(k\right)=\overset{¯}{\rho }\int dy\int_{0}^{2\pi }\frac{d\theta }{2\pi }\left(e^{i\mu k\cdot F(y,\hat{n}\left(\theta \right))}-1\right). \end{array}$$

Eq. **13** relies on an initial PDF p(v)=p(v)/(2π) and the property that θ satisfies angular translation invariance, i.e., the propagator $G_{v}$ depends on differences $\theta^{\prime }-\theta$ not absolute angles, which indeed holds for ABPs, RTPs, and AOUPs, but not for CAPs, as confirmed in simulations for Eq. **9**, see Fig. 1*B*. Note that evaluating Eq. **13** with Eq. **9** yields again $P(v_{x})$ of Eq. **11**, but with c=3.17|p|.

(c) In the adiabatic regime Eq. **7**, the tracer displacement statistics is obtained by solving an occupation time problem: Substituting $k_{u}=\Theta \left(\tau -u\right)k$ in Eq. **5** and using Eq. **7** yields the remaining average $\langle e^{i\mu \int_{0}^{\tau }duk\cdot F\left(Y_{u}\right)}\rangle_{Y_{0}=y}$, which corresponds to the characteristic function of the generalized occupation time $\mu \int_{0}^{\tau }duF\left(Y_{u}\right)$. This connection implies that for τ≫1, the statistics depends on the recurrence properties of $Y_{t}$ and is thus strongly affected by dimensionality.

(d) For the second-order approximation Eq. **8** a Feynman–Kac (FK) equation can be derived that assumes a generic form in any dimension and for any forces when the swimmer velocity ${\dot{Y}}_{t}$ is a Markov process. The quantity of interest is the average $Q_{\tau }\left(y;k\right)$ defined by:

[14] $$\begin{array}{c} Q_{\tau }=\langle e^{i\mu \int_{0}^{\tau }duk\cdot F\left(Y_{u}\right)-i\mu^{2}\int_{0}^{\tau }du\int_{0}^{u}dsk\cdot \left(\nabla F{\left(Y_{u}\right)}^{T}F\left(Y_{s}\right)\right)}\rangle , \end{array}$$

conditional on $Y_{0}=y$. As shown in *SI Appendix*, 8, $Q_{\tau }$ of Eq. **14** can be determined as:

[15] $$\begin{array}{c} Q_{\tau }\left(y;k\right)=\int dy^{\prime }dv^{\prime }dvdap\left(v\right)e^{ik\cdot a}{\tilde{Q}}_{\tau }\left(y^{\prime },v^{\prime },a|y,v;k\right), \end{array}$$

where ${\tilde{Q}}_{\tau }$ is the solution of the forward FK-type equation

[16] $$\begin{array}{c} \frac{\partial }{\partial \tau }{\tilde{Q}}_{\tau }=L{\tilde{Q}}_{\tau }-\mu F\left(y^{\prime }\right)\cdot \nabla_{a}{\tilde{Q}}_{\tau }-i\mu k\cdot \left(\nabla F{\left(y^{\prime }\right)}^{T}a\right){\tilde{Q}}_{\tau }, \end{array}$$

with initial condition ${\tilde{Q}}_{0}\left(y^{\prime },v^{\prime },a|y,v;k\right)=\delta \left(y^{\prime }-y\right)\delta \left(v^{\prime }-v\right)$δ(a) and L given as the generator of the swimmer dynamics. Eq. **16** yields controlled approximations of the tracer statistics beyond the adiabatic regime. For straight-line swimmer motion, an exact solution of Eq. **16** is derived for any F, see *SI Appendix*, 8A. For RTPs, L contains a term proportional in the tumble rate ω, which can be used as a perturbation to expand around the straight-line motion leading to exact iterative equations for $Q_{\tau }$ at any order in ω, see *SI Appendix*, 8B. Similar approximations might be possible for ABPs, AOUPs, and CAPs for small diffusivities or large persistence times.

In summary, a first-principles derivation of the coarse-grained tracer process of Eq. **1** has been presented. Applications and extensions of the framework could concern: i) nonspherical swimmer shapes (13), which can be readily incorporated by a suitable choice of F; ii) nonspherical tracer shapes (14), which would require to add the time evolution of the tracer’s orientational degrees of freedom to Eq. **1**, but should leave the resulting Poisson statistics unaffected; and iii) multiple tracer particles, which could be treated within a unified colored noise approximation (15, 16), see *SI Appendix*, 9. Conceptually, it would be highly interesting to explore the connection between the formalism and that of GLEs (6–8), which both describe an effective non-Markovian evolution of the tracer due to the interaction with the environment.

## Supplementary Material

Appendix 01 (PDF)

## Data Availability

All study data are included in the article and/or *SI Appendix*. A.B. designed research; performed research; analyzed data; and wrote the paper. The authors declare no competing interest.
