# Supplementary material for: Universal Poisson statistics of a passive tracer diffusing in dilute active suspensions
Source: Proc Natl Acad Sci U S A. 2023 Dec 4;120(50):e2308226120. doi: 10.1073/pnas.2308226120 (PMC10723115; doi:10.1073/pnas.2308226120)
Supplement: Supplementary file 1 — Appendix 01 (PDF) [file pnas.2308226120.sapp.pdf]

Supporting Information for

Universal Poisson statistics of a passive tracer diffusing in dilute active suspensions

Adrian Baule

E-mail: a.baule@qmul.ac.uk

This PDF file includes:

Supporting text
SI References

Supporting Information Text

1. Dimensionless units

Considering an equation of motion as in Eq. [1] with dimensional units, I assume that the force can be expressed in the form $\mathbf{F}(\mathbf{x}) = F_0\tilde{\mathbf{F}}(\mathbf{x}/l_0)$, where F_0 sets the scale of the force, l_0 is a length scale associated with the force, e.g., its interaction range, and $\tilde{\mathbf{F}}$ is dimensionless. The swimmer dynamics typically contains a characteristic velocity $v_{\rm A}$, e.g., the constant speed for active Brownian particles or run-and-tumble particles, such that a time scale can be defined as $\tau_0 = l_0/v_{\rm A}$. Introducing dimensionless positions and time as $\tilde{\mathbf{X}} = \mathbf{X}/l_0$, $\tilde{\mathbf{Y}} = \mathbf{Y}/l_0$, $\tilde{t} = t/\tau_0$ yields in Eq. [1]

$$\begin{aligned} \frac{\mathrm{d}\tilde{\mathbf{X}}(\tilde{t})}{\mathrm{d}\tilde{t}} &= \frac{\tau_0}{l_0}\mu F_0\,\tilde{\mathbf{F}}(\tilde{\mathbf{X}}(\tilde{t}) - \tilde{\mathbf{Y}}(\tilde{t})) \\ &= \tilde{\mu}\,\tilde{\mathbf{F}}(\tilde{\mathbf{X}}(\tilde{t}) - \tilde{\mathbf{Y}}(\tilde{t})), \end{aligned} \qquad \text{[S1]}$$

where the dimensionless mobility coefficient $\tilde{\mu}$ is defined as

$$\tilde{\mu} = \frac{\mu F_0}{v_{\rm A}}. \qquad \text{[S2]}$$

Dropping the tildes yields Eq. [1] in dimensionless form.

A. Dilute regime in experiments. For suspensions of swimming microorganisms, the characteristic length scale would be set by the size of the swimmers, which corresponds to their hard-core interaction range, or some other measure of the swimmer geometry. This convention can also be applied when the force is long-range without a well-defined interaction range (1). As a consequence, the dilute condition $\bar{\rho} \ll 1$ in dimensionless units becomes $\bar{\rho}\, l_0^d \ll 1$ (in d dimensions), which is approximately equal to the volume/surface concentration φ_d and can be easily checked for experimental situations. One can then verify that dilute conditions have indeed been realized in many experiments on swimming microorganisms and self-propelled colloid suspensions from the literature. The following list is by no means exhaustive and denotes the approximate range of φ_d for the different experiments performed:

For *Escherichia coli* bacteria:

- *Wu & Libchaber* (2): $\varphi_3 \approx 0.01 - 0.1$
- *Gachelin et al.:* $\varphi_3 \approx 0.001 - 0.03$ (3)
- *Lagarde et al.:* $\varphi_3 \approx 10^{-5} - 0.2$ (4)
- *Kamdar et al.:* $\varphi_3 \approx 4 \times 10^{-4}$ (5)

For comparison, *Escherichia coli* concentrations in the wild have been measured considerably lower with $\varphi_3 \ll 10^{-5}$ (6).

For *Chlamydomonas reinhardtii* algae:

- *Leptos et al.:* $\varphi_3 \approx 0.004 - 0.02$ (7)
- *Kurtuldu et al.:* $\varphi_2 \approx 0.007 - 0.07$ (8)
- *Jeanneret et al.:* $\varphi_3 \approx 10^{-4} - 5 \times 10^{-3}$ (9)
- *Kurihara et al.:* $\varphi_2 \approx 10^{-3} - 2 \times 10^{-2}$ (10)

For *self-propelled Janus colloids*:

- *Wang et al.:* $\varphi_2 \approx 10^{-4}$ (11)
- *Sachs et al.:* $\varphi_3 \approx 10^{-4} - 10^{-3}$ (12)
- *Singh et al.:* $\varphi_2 \approx 0.06$ (13)

B. Regime of small mobility coefficient. Since μF_0 is the typical velocity of the tracer induced by the interaction with a swimmer, we see that the (dimensionless) mobility coefficient $\tilde{\mu}$ of Eq. [S2] is given as the ratio of the velocities characterising the tracer and swimmer dynamics. As a consequence, $\tilde{\mu}$ is small when the characteristic velocity of the swimmers is much larger than that of the tracer. Under this condition an expansion in $\tilde{\mu}$, used in the main text to derive analytical predictions, is justified. Note that other coarse-graining schemes rely on very similar assumptions. The adiabatic perturbation theory used in (14), e.g., requires likewise that the diffusive relaxation of the active bath is much faster than the response of the tracer.

One can further verify that $\tilde{\mu}$ is typically small for tracers of a few μm size interacting with swimming microorganisms or active colloids in suspension. The dimensional mobility can be calculated from Stokes law as $\mu = (6\pi\, a\, \nu)^{-1}$, where a denotes the tracer radius and ν the shear viscosity of the liquid ($\approx 8.9 \times 10^{-4}$Js/m^3 for water at room temperature). This result for the mobility is valid at low Reynolds numbers and for dilute suspensions of swimmers. Assuming a tracer radius of $a \approx 5\mu$m, we obtain $\mu \approx 1.2 \times 10^7$m^2/(Js). Moreover, we have $v_{\rm A} \approx 1.5 \times 10^{-5}$m/s for *Escherichia coli* (4), $v_{\rm A} \approx 8 \times 10^{-5}$m/s

for *Chlamydomonas reinhardtii* (10), and $v_{\rm A} \approx 8 \times 10^{-6}$m/s for Janus colloids (12). Characteristic force scales range from $F_0 \approx 10^{-13}$N for electrostatic (15) and mechanical interactions (16) to $F_0 \approx 10^{-18}$N for hydrodynamic interactions (17), while phoretic interactions are intermediate in strength. This yields throughout the range of realistic interactions and for all three types of active suspensions from Eq. [S2]

$$\tilde{\mu} = \frac{F_0}{6\pi\, a\, \nu\, v_{\rm A}} < 0.15.$$

These estimates confirm that the regime of small $\tilde{\mu}$ used in the theory is indeed relevant in many practical scenarios.

2. Correlation functions of the density field

In order to derive Eq. [4], I first determine the 2-point density correlation function for finite N, V. Using the definition of the microscopic density field

$$\rho(\mathbf{x}, t) = \sum_{i=1}^{N} \delta(\mathbf{x} - \mathbf{Y}_i(t)) \qquad \text{[S3]}$$

and the fact that the swimmers are non-interacting, we obtain

$$\begin{aligned} \langle \rho(\mathbf{x}_1, t_1)\rho(\mathbf{x}_2, t_2)\rangle &= \sum_{i=1}^{N}\sum_{j=1}^{N} \langle \delta(\mathbf{x}_1 - \mathbf{Y}_i(t_1))\delta(\mathbf{x}_2 - \mathbf{Y}_j(t_2))\rangle \\ &= N\,\langle \delta(\mathbf{x}_1 - \mathbf{Y}(t_1))\delta(\mathbf{x}_2 - \mathbf{Y}(t_2))\rangle \\ &\quad + N(N-1)\,\langle \delta(\mathbf{x}_1 - \mathbf{Y}(t_1))\rangle\,\langle \delta(\mathbf{x}_2 - \mathbf{Y}(t_2))\rangle\,. \end{aligned} \qquad \text{[S4]}$$

The average is taken with respect to the initial positions, which are assumed uniform in the volume V, and the stochastic process underlying the dynamics of $\mathbf{Y}$. In the second term, the average over the initial positions yields

$$\begin{aligned} \langle \delta(\mathbf{x}_1 - \mathbf{Y}(t_1))\rangle &= \frac{1}{V}\int \mathrm{d}\mathbf{y}\,\langle \delta(\mathbf{x}_1 - \mathbf{Y}(t_1))\rangle_{\mathbf{Y}(0)=\mathbf{y}} \\ &= \frac{1}{V}\int \mathrm{d}\mathbf{y}\, G(\mathbf{x}_1 - \mathbf{y}, t_1) \\ &= \frac{1}{V}. \end{aligned} \qquad \text{[S5]}$$

which is a simple consequence of the translation invariance of the propagator $G(\mathbf{x}_1 - \mathbf{y}, t_1) = \langle \delta(\mathbf{x}_1 - \mathbf{Y}(t_1))\rangle_{\mathbf{Y}(0)=\mathbf{y}}$. As a consequence

$$\langle \rho(\mathbf{x}_1, t_1)\rho(\mathbf{x}_2, t_2)\rangle = \frac{N}{V}\int \mathrm{d}\mathbf{y}\,\langle \delta(\mathbf{x}_1 - \mathbf{Y}(t_1))\delta(\mathbf{x}_2 - \mathbf{Y}(t_2))\rangle_{\mathbf{Y}(0)=\mathbf{y}} + \frac{N(N-1)}{V^2} \qquad \text{[S6]}$$

and introducing the swimmer number density $\bar{\rho} = N/V$ leads to the result in the thermodynamic limit

$$\langle \rho(\mathbf{x}_1, t_1)\rho(\mathbf{x}_2, t_2)\rangle = \bar{\rho}\int \mathrm{d}\mathbf{y}\,\langle \delta(\mathbf{x}_1 - \mathbf{Y}(t_1))\delta(\mathbf{x}_2 - \mathbf{Y}(t_2))\rangle_{\mathbf{Y}(0)=\mathbf{y}} + \bar{\rho}^2. \qquad \text{[S7]}$$

For the 3-point correlation function we obtain likewise

$$\begin{aligned} \langle \rho(\mathbf{x}_1, t_1)\rho(\mathbf{x}_2, t_2)\rho(\mathbf{x}_3, t_3)\rangle &= \sum_{i=1}^{N}\sum_{j=1}^{N}\sum_{l=1}^{N} \langle \delta(\mathbf{x}_1 - \mathbf{Y}_i(t_1))\delta(\mathbf{x}_2 - \mathbf{Y}_j(t_2))\delta(\mathbf{x}_3 - \mathbf{Y}_l(t_3))\rangle \\ &= N\,\langle \delta(\mathbf{x}_1 - \mathbf{Y}(t_1))\delta(\mathbf{x}_2 - \mathbf{Y}(t_2))\delta(\mathbf{x}_3 - \mathbf{Y}(t_3))\rangle \\ &\quad + N(N-1)\,\langle \delta(\mathbf{x}_1 - \mathbf{Y}(t_1))\rangle\,\langle \delta(\mathbf{x}_2 - \mathbf{Y}(t_2))\delta(\mathbf{x}_3 - \mathbf{Y}(t_3))\rangle \\ &\quad + N(N-1)\,\langle \delta(\mathbf{x}_2 - \mathbf{Y}(t_2))\rangle\,\langle \delta(\mathbf{x}_1 - \mathbf{Y}(t_1))\delta(\mathbf{x}_3 - \mathbf{Y}(t_3))\rangle \\ &\quad + N(N-1)\,\langle \delta(\mathbf{x}_3 - \mathbf{Y}(t_3))\rangle\,\langle \delta(\mathbf{x}_2 - \mathbf{Y}(t_2))\delta(\mathbf{x}_1 - \mathbf{Y}(t_1))\rangle \\ &\quad + N(N-1)(N-2)\,\langle \delta(\mathbf{x}_1 - \mathbf{Y}(t_1))\rangle\,\langle \delta(\mathbf{x}_2 - \mathbf{Y}(t_2))\rangle\,\times \\ &\qquad \langle \delta(\mathbf{x}_3 - \mathbf{Y}(t_3))\rangle\,. \end{aligned} \qquad \text{[S8]}$$

Taking the thermodynamic limit and considering Eq. [S5] leads to

$$\begin{aligned}\langle\rho(\mathbf{x}_1,t_1)\rho(\mathbf{x}_2,t_2)\rho(\mathbf{x}_3,t_3)\rangle &= \bar{\rho}\int \mathrm{d}\mathbf{y}\,\langle\delta(\mathbf{x}_1-\mathbf{Y}(t_1))\delta(\mathbf{x}_2-\mathbf{Y}(t_2))\delta(\mathbf{x}_3-\mathbf{Y}(t_3))\rangle_{\mathbf{Y}(0)=\mathbf{y}}\\ &+\bar{\rho}^2\left\{\int \mathrm{d}\mathbf{y}\,\langle\delta(\mathbf{x}_2-\mathbf{Y}(t_2))\delta(\mathbf{x}_3-\mathbf{Y}(t_3))\rangle_{\mathbf{Y}(0)=\mathbf{y}}\right.\\ &+\int \mathrm{d}\mathbf{y}\,\langle\delta(\mathbf{x}_1-\mathbf{Y}(t_3))\delta(\mathbf{x}_3-\mathbf{Y}(t_3))\rangle_{\mathbf{Y}(0)=\mathbf{y}}\\ &\left.+\int \mathrm{d}\mathbf{y}\,\langle\delta(\mathbf{x}_2-\mathbf{Y}(t_2))\delta(\mathbf{x}_1-\mathbf{Y}(t_1))\rangle_{\mathbf{Y}(0)=\mathbf{y}}\right\}+\bar{\rho}^3\end{aligned}\qquad [S9]$$

It is then easy to see that for the n-point function the term to lowest order in $\bar{\rho}$ contains the n-point position PDF of a single swimmer conditioned on $\mathbf{Y}(0)=\mathbf{y}$, i.e., it contains only single-particle self-correlations. This is also the only fully connected contribution to the n-point function, while all higher orders in $\bar{\rho}$ are at least partially disconnected. To first order in $\bar{\rho}$ the n-point function can thus be approximated as in Eq. [4].

3. Evaluating the averages in the series expansion

Expanding Eq. [3] with $A[\mathbf{X}]$ given by $A[\mathbf{X}]=e^{i\int_0^t \mathrm{d}u\,\mathbf{k}(u)\cdot\dot{\mathbf{X}}(u)}$ into a perturbative series in μ yields a series representation of the CF Eq. [2] in the form

$$\psi_{\dot{\mathbf{X}}}[\mathbf{k}]=\left\langle e^{i\int_0^t \mathrm{d}u\,\mathbf{k}(u)\cdot\dot{\mathbf{X}}(u)}\right\rangle_0+\sum_{n=1}^{\infty}\frac{(i\mu)^n}{n!}\left\langle e^{i\int_0^t \mathrm{d}u\int \mathrm{d}\mathbf{x}\,\mathbf{k}(u)\cdot\dot{\mathbf{X}}(u)}\left\langle\prod_{i=1}^{n}\int_0^t \mathrm{d}s_i\,\mathbf{g}(s_i)\cdot\mathbf{F}(\mathbf{x}_i-\mathbf{X}(s_i))\rho(\mathbf{x}_i,s_i)\right\rangle\right\rangle_0,\qquad [S10]$$

where I define

$$\Big\langle ...\Big\rangle_0=\int\mathcal{D}\left[\frac{\mathbf{g}}{2\pi}\right]\int\mathcal{D}[\mathbf{X}]...\delta(\mathbf{X}(0))e^{i\int_0^t \mathrm{d}s\,\mathbf{g}(s)\cdot\dot{\mathbf{X}}(s)},\qquad [S11]$$

and the remaining average $\langle...\rangle$ is taken with respect to the swimmer dynamics. Using Eq. [4] for the n-point functions of $\rho(\mathbf{x},t)$ in the dilute regime, we obtain then

$$\begin{aligned}\psi_{\dot{\mathbf{X}}}[\mathbf{k}] &\approx \left\langle e^{i\int_0^t \mathrm{d}u\,\mathbf{k}(u)\cdot\dot{\mathbf{X}}(u)}\right\rangle_0+\bar{\rho}\int\mathrm{d}\mathbf{y}\left\langle\left\langle e^{i\int_0^t \mathrm{d}u\,\mathbf{k}(u)\cdot\dot{\mathbf{X}}(u)}\sum_{n=1}^{\infty}\frac{(i\mu)^n}{n!}\prod_{i=1}^{n}\int_0^t\mathrm{d}s_i\,\mathbf{g}(s_i)\cdot\mathbf{F}(\mathbf{Y}(s_i)-\mathbf{X}(s_i))\right\rangle_0\right\rangle_{\mathbf{Y}(0)=\mathbf{y}}\\ &= 1+\bar{\rho}\int\mathrm{d}\mathbf{y}\left\langle\sum_{m=1}^{\infty}\frac{i^m}{m!}\left(\int_0^t\mathrm{d}s\,\mathbf{k}(s)\cdot\mathbf{f}^*[\mathbf{Y}(s)]\right)^m\right\rangle_{\mathbf{Y}(0)=\mathbf{y}}\end{aligned}\qquad [S12]$$

after evaluating explicitly the averages $\langle...\rangle_0$ in each term of the series. The main result Eq. [5] now follows directly from Eq. [S12].

The last step in Eq. [S12] is due to the identity

$$\left\langle e^{i\int_0^t \mathrm{d}u\,\mathbf{k}(u)\cdot\dot{\mathbf{X}}(u)}\sum_{n=1}^{\infty}\frac{(i\mu)^n}{n!}\prod_{i=1}^{n}\int_0^t\mathrm{d}s_i\,\mathbf{g}(s_i)\cdot\mathbf{F}(\mathbf{Y}(s_i)-\mathbf{X}(s_i))\right\rangle_0=\sum_{m=1}^{\infty}\frac{i^m}{m!}\left(\int_0^t\mathrm{d}s\,\mathbf{k}(s)\cdot\mathbf{f}^*[\mathbf{Y}(s)]\right)^m,\qquad [S13]$$

which is the key result that allows the resummation of the series in Eq. [S10] to first order in $\bar{\rho}$ leading to Eq. [S12]. In addition, we have

$$\left\langle e^{i\int_0^t \mathrm{d}u\,\mathbf{k}(u)\cdot\dot{\mathbf{X}}(u)}\right\rangle_0=1,\qquad [S14]$$

since $\langle...\rangle_0$ simply constrains the dynamics of $\mathbf{X}(t)$ to the constant motion $\dot{\mathbf{X}}(t)=\mathbf{0}$ with initial tracer position $\mathbf{X}(0)=\mathbf{0}$. In Eq. [S13], the functional $\mathbf{f}^*[\mathbf{Y}]$ is obtained from the two-body interaction between the tracer and a single swimmer following trajectory $\mathbf{Y}(t)$

$$\dot{\mathbf{X}}(t)=\mu\,\mathbf{F}(\mathbf{Y}(t)-\mathbf{X}(t)).\qquad [S15]$$

If we consider $\mathbf{Y}$ as a given function prescribing the time dependence in the force and denote the solution of Eq. [S15] as $\mathbf{X}^*$, $\mathbf{f}^*[\mathbf{Y}]$ is given as

$$\mathbf{f}^*[\mathbf{Y}]=\frac{\mathrm{d}}{\mathrm{d}t}\mathbf{X}^*(t).\qquad [S16]$$

An explicit expression for $\mathbf{f}^*[\mathbf{Y}]$ can be derived from a fixed-point (Picard) iteration considering Eq. [S15] in integrated form

$$\mathbf{X}(t) = \mu \int_0^t \mathrm{d}s\, \mathbf{F}(\mathbf{Y}(s) - \mathbf{X}(s)), \tag{S17}$$

where $\mathbf{X}(0) = \mathbf{0}$. Up to second order we have then

$$\mathbf{X}^{*,2}(t) \;=\; \mu \int_0^t \mathrm{d}s\, \mathbf{F}\left(\mathbf{Y}(s) - \mu \int_0^s \mathrm{d}s'\, \mathbf{F}\left(\mathbf{Y}(s')\right)\right) \tag{S18}$$

and thus

$$\mathbf{f}^*[\mathbf{Y}] \approx \mu\, \mathbf{F}\left(\mathbf{Y}(t) - \mu \int_0^t \mathrm{d}s\, \mathbf{F}\left(\mathbf{Y}(s)\right)\right). \tag{S19}$$

Taylor expansion in orders of μ then yields the approximations in Eqs. [7,8]. Before I establish Eq. [S13] by evaluating the averages order-by-order in μ, I present a heuristic argument for its validity.

A. Heuristic proof based on the MSRJD-formalism. Note that the lhs of Eq. [S13] can be written as

$$\begin{aligned}\left\langle e^{i\int_0^t \mathrm{d}u\, \mathbf{k}(u)\cdot\dot{\mathbf{X}}(u)} \sum_{n=1}^{\infty} \frac{(i\mu)^n}{n!} \prod_{i=1}^{n} \int_0^t \mathrm{d}s_i\, \mathbf{g}(s_i)\cdot \mathbf{F}(\mathbf{Y}(s_i) - \mathbf{X}(s_i))\right\rangle_0 &= \left\langle e^{i\int_0^t \mathrm{d}u\, \mathbf{k}(u)\cdot\dot{\mathbf{X}}(u)} \left(e^{i\mu\int_0^t \mathrm{d}s\, \mathbf{g}(s)\cdot\mathbf{F}(\mathbf{Y}(s)-\mathbf{X}(s))} - 1\right)\right\rangle_0 \\ &= \left\langle e^{i\int_0^t \mathrm{d}u\, \mathbf{k}(u)\cdot\dot{\mathbf{X}}(u) + i\mu\int_0^t \mathrm{d}s\, \mathbf{g}(s)\cdot\mathbf{F}(\mathbf{Y}(s)-\mathbf{X}(s))}\right\rangle_0 - 1\end{aligned} \tag{S20}$$

The remaining average

$$\begin{aligned}\left\langle e^{i\int_0^t \mathrm{d}u\, \mathbf{k}(u)\cdot\dot{\mathbf{X}}(u) + i\mu\int_0^t \mathrm{d}s\, \mathbf{g}(s)\cdot\mathbf{F}(\mathbf{Y}(s)-\mathbf{X}(s))}\right\rangle_0 \;&=\; \int \mathcal{D}\left[\frac{\mathbf{g}}{2\pi}\right] \int \mathcal{D}[\mathbf{X}]\delta(\mathbf{X}(0)) e^{i\int_0^t \mathrm{d}u\, \mathbf{k}(u)\cdot\dot{\mathbf{X}}(u)} \times \\ &\quad e^{i\mu\int_0^t \mathrm{d}s\, \mathbf{g}(s)\cdot\mathbf{F}(\mathbf{Y}(s)-\mathbf{X}(s)) - i\int_0^t \mathrm{d}s\, \mathbf{g}(s)\cdot\dot{\mathbf{X}}(s)}\end{aligned} \tag{S21}$$

is nothing but the MSRJD expression for the average $\left\langle e^{i\int_0^t \mathrm{d}u\, \mathbf{k}(u)\cdot\dot{\mathbf{X}}(u)}\right\rangle$ constraining $\mathbf{X}(t)$ to the solution of the ODE Eq. [S15]. This implies that

$$\left\langle e^{i\int_0^t \mathrm{d}u\, \mathbf{k}(u)\cdot\dot{\mathbf{X}}(u) + i\mu\int_0^t \mathrm{d}s\, \mathbf{g}(s)\cdot\mathbf{F}(\mathbf{Y}(s)-\mathbf{X}(s))}\right\rangle_0 = e^{i\int_0^t \mathrm{d}u\, \mathbf{k}(u)\cdot\mathbf{f}^*[\mathbf{Y}(u)]} \tag{S22}$$

using Eq. [S16] and Eq. [S13] follows.

B. Proof based on the term-by-term evaluation of averages. In this section, I simplify the notation by considering only one dimension. The identity Eq. [S13] expresses a general connection between the averages over $\langle ... \rangle_0$ and the solution of an ODE. In general terms we consider the ODE

$$\dot{X}(t) = \mu\, F(t, X(t)), \qquad X(0) = x, \tag{S23}$$

where the explicit time-dependence in F stems in our original problem from the dependence on the swimmer trajectory $Y(t)$. I assume that the solution $X^*(t)$ of Eq. [S23] can be expressed as a power-series in μ

$$X^*(t) = x + \sum_{n=1}^{\infty} \mu^n \int_0^t \mathrm{d}s\, \phi_n(s, x). \tag{S24}$$

The equivalent identity to Eq. [S13] is then

$$\left\langle e^{i\int_0^t \mathrm{d}u\, z(u)\dot{X}(u)} \sum_{n=1}^{\infty} \frac{(i\mu)^n}{n!} \prod_{i=1}^{n} \int_0^t \mathrm{d}s_i\, g(s_i) F(s_i, X(s_i))\right\rangle_0 \;=\; \sum_{m=1}^{\infty} \frac{i^m}{m!} \left(\int_0^t \mathrm{d}s\, z(s) \sum_{n=1}^{\infty} \mu^n \phi_n(s, x)\right)^m, \tag{S25}$$

which will be established order-by-order in μ.

B.1. The functions ϕ_n. The series representation of Eq. [S24] can be obtained from a Picard iteration of Eq. [S23] and subsequent Taylor expansion around x. For the first three orders in μ, e.g., one requires the third Picard iteration

$$X^{*,3}(t) = \mu \int_0^t \mathrm{d}s\, F\left(s, x + \mu \int_0^s \mathrm{d}s'\, F\left(s', x + \mu \int_0^{s'} \mathrm{d}s''\, F(s'', x)\right)\right) \tag{S26}$$

and the base functions ϕ_n in Eq. [S24] are determined as

$$\phi_1(s,x) = F(s,x) \tag{S27}$$

$$\phi_2(s,x) = \partial_x F(s,x) \int_0^s \mathrm{d}s'\, F(s',x) \tag{S28}$$

$$\begin{aligned} \phi_3(s,x) &= \partial_x F(s,x) \int_0^s \mathrm{d}s'\, \partial_x F(s',x) \int_0^{s'} \mathrm{d}s''\, F(s'',x) \\ &\quad + \frac{1}{2}\partial_x^2 F(s,x) \int_0^s \mathrm{d}s'\, F(s',x) \int_0^s \mathrm{d}s''\, F(s'',x) \end{aligned} \tag{S29}$$

Crucially, I claim that the nth order function ϕ_n can also be obtained as a path integral average in the following way

$$\phi_n(s_n,x) = \frac{1}{(n-1)!} \int_0^t \mathrm{d}s_1 ... \int_0^t \mathrm{d}s_{n-1} \left\langle \frac{\delta}{\delta \dot{X}(s_1)} \cdots \frac{\delta}{\delta \dot{X}(s_{n-1})} F(s_1, X(s_1)) \cdots F(s_{n-1}, X(s_{n-1})) F(s_n, X(s_n)) \right\rangle_0, \tag{S30}$$

where the brackets again indicate an average with respect to paths constrained to $X(t) = x$. I verify the validity of Eq. [S30] for each n separately noting that

$$\frac{\delta}{\delta \dot{X}(s_j)} F(s_l, X(s_l)) = \int_0^{s_l} \mathrm{d}u\, \delta(u - s_j) \partial_x F(s_l, X(s_l)) = 0 \tag{S31}$$

when $j = l$ (due to the standard path integral discretization used) or when $s_l < s_j$. Evaluating the rhs of Eq. [S30] up to $n = 3$ yields

$$\phi_1(s,x) = \langle F(s, X(s)) \rangle_0 = F(s,x) \tag{S32}$$

$$\begin{aligned} \phi_2(s_2,x) &= \int_0^t \mathrm{d}s_1 \left\langle \frac{\delta}{\delta \dot{X}(s_1)} F(s_1, X(s_1)) F(s_2, X(s_2)) \right\rangle_0 \\ &= \int_0^t \mathrm{d}s_1 \Theta(s_2 - s_1) \langle F(s_1, X(s_1)) \partial_x F(s_2, X(s_2)) \rangle_0 \\ &= \int_0^{s_2} \mathrm{d}s_1 F(s_1, x) \partial_x F(s_2, x) \end{aligned} \tag{S33}$$

$$\begin{aligned} \phi_3(s_3,x) &= \frac{1}{2} \int_0^t \mathrm{d}s_1 \int_0^t \mathrm{d}s_2 \left\langle \frac{\delta}{\delta \dot{X}(s_1)} \frac{\delta}{\delta \dot{X}(s_2)} F(s_1, X(s_1)) F(s_2, X(s_2) F(s_3, X(s_3))) \right\rangle_0 \\ &= \frac{1}{2} \int_0^t \mathrm{d}s_1 \int_0^t \mathrm{d}s_2 \Big\{ \Theta(s_3 - s_2)\Theta(s_3 - s_1) \left\langle F(s_1, X(s_1)) F(s_2, X(s_2) \partial_x^2 F(s_3, X(s_3))) \right\rangle_0 \\ &\quad + \Theta(s_3 - s_2)\Theta(s_2 - s_1) \langle F(s_1, X(s_1)) \partial_x F(s_2, X(s_2)) \partial_x F(s_3, X(s_3)) \rangle_0 \\ &\quad + \Theta(s_3 - s_1)\Theta(s_1 - s_2) \langle \partial_x F(s_1, X(s_1)) F(s_2, X(s_2) \partial_x F(s_3, X(s_3))) \rangle_0 \Big\} \\ &= \frac{1}{2} \int_0^{s_3} \mathrm{d}s_2 \int_0^{s_3} \mathrm{d}s_1 F(s_1,x) F(s_2,x) \partial_x^2 F(s_3,x) + \int_0^{s_3} \mathrm{d}s_2 \int_0^{s_2} \mathrm{d}s_1 F(s_1,x) \partial_x F(s_2,x) \partial_x F(s_3,x) \end{aligned} \tag{S34}$$

which agrees with the results for ϕ_n based on the Picard iteration. It is straightforward but cumbersome to verify that Eq. [S30] also holds for larger n.

B.2. Order-by-order evaluation of Eq. [S25]. I express the terms on the rhs of Eq. [**S25**] as a series in orders of μ and establish the equality for each order separately. Considering orders up to μ^3, the following equalities need to be shown:

$$\mu: \qquad i\int_0^t \mathrm{d}s \left\langle e^{i\int_0^t \mathrm{d}u\, z(u)\dot{X}(u)} g(s)F(s,X(s))\right\rangle_0 = i\int_0^t \mathrm{d}s\, z(s)\phi_1(s,x) \tag{S35}$$

$$\begin{aligned}\mu^2: \qquad & -\frac{1}{2}\int_0^t \mathrm{d}s_1\int_0^t \mathrm{d}s_2 \left\langle e^{i\int_0^t \mathrm{d}u\, z(u)\dot{X}(u)} g(s_1)g(s_2)F(s_1,X(s_1))F(s_2,X(s_2))\right\rangle_0 \\ & = i\int_0^t \mathrm{d}s\, z(s)\phi_2(s,x) - \frac{1}{2}\int_0^t \mathrm{d}s_1\int_0^t \mathrm{d}s_2\, z(s_1)z(s_2)\phi_1(s_1,x)\phi_1(s_2,x)\end{aligned} \tag{S36}$$

$$\begin{aligned}\mu^3: \qquad & -\frac{i}{6}\int_0^t \mathrm{d}s_1\int_0^t \mathrm{d}s_2\int_0^t \mathrm{d}s_3 \left\langle e^{i\int_0^t \mathrm{d}u\, z(u)\dot{X}(u)} g(s_1)g(s_2)g(s_3)F(s_1,X(s_1))F(s_2,X(s_2))F(s_3,X(s_3))\right\rangle_0 \\ & = i\int_0^t \mathrm{d}s\, z(s)\phi_3(s,x) - \int_0^t \mathrm{d}s_1\int_0^t \mathrm{d}s_2\, z(s_1)z(s_2)\phi_1(s_1,x)\phi_2(s_2,x) \\ & \quad -\frac{i}{6}\int_0^t \mathrm{d}s_1\int_0^t \mathrm{d}s_2\int_0^t \mathrm{d}s_3\, z(s_1)z(s_2)z(s_3)\phi_1(s_1,x)\phi_1(s_2,x)\phi_1(s_3,x)\end{aligned} \tag{S37}$$

Considering Eq. [**S35**], we have

$$\begin{aligned} i\int_0^t \mathrm{d}s \left\langle e^{i\int_0^t \mathrm{d}u\, z(u)\dot{X}(u)} g(s)F(s,X(s))\right\rangle_0 &= \int_0^t \mathrm{d}s \left\langle \frac{\delta}{\delta \dot{X}(s)} e^{i\int_0^t \mathrm{d}u\, z(u)\dot{X}(u)} F(s,X(s))\right\rangle_0 \\ &= i\int_0^t \mathrm{d}s\, z(s)F(s,x) \\ &= i\int_0^t \mathrm{d}s\, z(s)\phi_1(s,x)\end{aligned} \tag{S38}$$

using partial integration on the path weight in the first step and subsequently Eqs. (S31,S27).

Considering Eq. [**S36**], we have

$$\begin{aligned} & -\frac{1}{2}\int_0^t \mathrm{d}s_1\int_0^t \mathrm{d}s_2 \left\langle e^{i\int_0^t \mathrm{d}u\, z(u)\dot{X}(u)} g(s_1)g(s_2)F(s_1,X(s_1))F(s_2,X(s_2))\right\rangle_0 \\ & = \frac{1}{2}\int_0^t \mathrm{d}s_1\int_0^t \mathrm{d}s_2 \left\langle \frac{\delta}{\delta \dot{X}(s_1)}\frac{\delta}{\delta \dot{X}(s_2)} e^{i\int_0^t \mathrm{d}u\, z(u)\dot{X}(u)} F(s_1,X(s_1))F(s_2,X(s_2))\right\rangle_0\end{aligned} \tag{S39}$$

The combinatorics of the higher order partial derivatives becomes quickly tedious. Here, the situation is simplified since the $s_{1,2}$ variables are indistinguishable and we can use the general formula

$$\frac{\delta^n}{\delta \dot{X}(s_1)\cdots\delta \dot{X}(s_n)} h_\mathrm{a}(s_1,...,s_n)h_\mathrm{b}(s_1,...,s_n) = \sum_{l=0}^n \binom{n}{l} \delta^l h_\mathrm{a}\, \delta^{n-l} h_\mathrm{b}, \tag{S40}$$

where the shorthand notation $\delta^l h_\mathrm{a} = \frac{\delta^l}{\delta \dot{X}(s_1)\cdots\delta \dot{X}(s_l)} h_\mathrm{a}(s_1,...,s_l)$ is used with $\delta^0 h_\mathrm{a} = h_\mathrm{a}$. This yields

$$\begin{aligned} & \frac{1}{2}\int_0^t \mathrm{d}s_1\int_0^t \mathrm{d}s_2 \left\langle \frac{\delta}{\delta \dot{X}(s_1)}\frac{\delta}{\delta \dot{X}(s_2)} e^{i\int_0^t \mathrm{d}u\, z(u)\dot{X}(u)} F(s_1,X(s_1))F(s_2,X(s_2))\right\rangle_0 \\ & = \frac{1}{2}\int_0^t \mathrm{d}s_1\int_0^t \mathrm{d}s_2 \left\{ (-1)z(s_1)z(s_2)\left\langle F(s_1,X(s_1))F(s_2,X(s_2))\right\rangle_0 \right. \\ & \quad + i\, z(s_2)\left\langle \frac{\delta}{\delta \dot{X}(s_1)} F(s_1,X(s_1))F(s_2,X(s_2))\right\rangle_0 \\ & \quad \left. + \left\langle \frac{\delta}{\delta \dot{X}(s_1)}\frac{\delta}{\delta \dot{X}(s_2)} F(s_1,X(s_1))F(s_2,X(s_2))\right\rangle_0 \right\} \\ & = -\frac{1}{2}\int_0^t \mathrm{d}s_1\int_0^t \mathrm{d}s_2\, z(s_1)z(s_2)F(s_1,x)F(s_2,x) + i\int_0^t \mathrm{d}s\, z(s)\phi_2(s,x),\end{aligned} \tag{S41}$$

confirming Eq. [**S36**]. In the first step, I have used the factorization property of the average $\langle f_\mathrm{a}(X(s_1))f_\mathrm{b}(X(s_2))\rangle_0 = f_\mathrm{a}(x)\langle f_\mathrm{b}(X(s_2))\rangle_0$. In the last step, I have used Eq. [**S30**] with $n=2$ and Eq. [**S31**], which leads to the vanishing of the third term.

Considering Eq. [S37], we use again Eq. [S40] to calculate the functional derivatives

$$
\begin{aligned}
&-\frac{i}{6}\int_0^t \mathrm{d}s_1\int_0^t \mathrm{d}s_2\int_0^t \mathrm{d}s_3 \left\langle e^{i\int_0^t \mathrm{d}u\, z(u)\dot{X}(u)} g(s_1)g(s_2)g(s_3)F(s_1,X(s_1))F(s_2,X(s_2))F(s_3,X(s_3))\right\rangle_0\\
&=\frac{1}{6}\int_0^t \mathrm{d}s_1\int_0^t \mathrm{d}s_2\int_0^t \mathrm{d}s_3 \left\langle \frac{\delta}{\delta\dot{X}(s_1)}\frac{\delta}{\delta\dot{X}(s_2)}\frac{\delta}{\delta\dot{X}(s_3)} e^{i\int_0^t \mathrm{d}u\, z(u)\dot{X}(u)} F(s_1,X(s_1))F(s_2,X(s_2))F(s_3,X(s_3))\right\rangle_0\\
&=\frac{1}{6}\int_0^t \mathrm{d}s_1\int_0^t \mathrm{d}s_2\int_0^t \mathrm{d}s_3 \left\{(-i)z(s_1)z(s_2)z(s_3)F(s_1,x)F(s_2,x)F(s_3,x)\right.\\
&\quad -3\,z(s_2)z(s_3)\left\langle \frac{\delta}{\delta\dot{X}(s_1)}F(s_1,X(s_1))F(s_2,X(s_2))F(s_3,X(s_3))\right\rangle_0\\
&\quad \left.+\,3iz(s_3)\left\langle \frac{\delta}{\delta\dot{X}(s_1)}\frac{\delta}{\delta\dot{X}(s_2)}F(s_1,X(s_1))F(s_2,X(s_2))F(s_3,X(s_3)\right\rangle_0\right\}\\
&=\frac{1}{6}\int_0^t \mathrm{d}s_1\int_0^t \mathrm{d}s_2\int_0^t \mathrm{d}s_3 \left\{(-i)z(s_1)z(s_2)z(s_3)\phi_1(s_1,x)\phi_1(s_2,x)\phi_1(s_3,x)\right.\\
&\quad \left.-6\,z(s_2)z(s_3)F(s_1,x)\Theta(s_2-s_1)\partial_x F(s_2,x)F(s_3,x)+6iz(s_3)\phi_3(s_3,x)\right\}
\end{aligned}
\qquad \text{[S42]}
$$

using again Eqs. [S30,S31] and thus confirming Eq. [S37]. The correspondence for higher orders can be shown following the same calculation techniques.

4. The characteristic functional of the spatial Poisson process

The tracer dynamics can be expressed as a stochastic equation in the form

$$\dot{\mathbf{X}}_t = \sum_{\mathbf{y}\in\Phi}\mathbf{f}^*[\mathbf{Y}_t] \qquad \text{[S43]}$$

where Φ denotes a spatial Poisson process with intensity $\bar{\rho}$. This implies that the number of swimmers $\tilde{N}$ in a subvolume $\tilde{V}$ follows a Poisson distribution with intensity $\bar{\rho}$, i.e., $P(\tilde{N}=m)=\frac{(\bar{\rho}\tilde{V})^m}{m!}e^{-\bar{\rho}\tilde{V}}$, and their positions are uniformly distributed in $\tilde{V}$. In order to show that Eq. [S43] is equivalent to Eq. [5], I first assume a finite volume $\tilde{V}$. Substituting Eq. [S43] into Eq. [2] yields

$$
\begin{aligned}
\left\langle \exp\left\{i\int_0^t \mathbf{k}(s)\cdot\dot{\mathbf{X}}(s)\mathrm{d}s\right\}\right\rangle &= \left\langle \exp\left\{i\int_0^t \mathbf{k}(s)\cdot\sum_{\mathbf{y}\in\Phi}\mathbf{f}^*[\mathbf{Y}(s)]\mathrm{d}s\right\}\right\rangle\\
&= \sum_{m=0}^{\infty}P(\tilde{N}=m)\prod_{i=0}^{m}\frac{1}{\tilde{V}}\int_{\tilde{V}}\mathrm{d}\mathbf{y}\left\langle \exp\left\{i\int_0^t \mathbf{k}(s)\cdot\mathbf{f}^*[\mathbf{Y}(s)]\mathrm{d}s\right\}\right\rangle_{\mathbf{Y}(0)=\mathbf{y}}\\
&= \sum_{m=0}^{\infty}\frac{(\bar{\rho}\tilde{V})^m}{m!}e^{-\bar{\rho}\tilde{V}}\left(\frac{1}{\tilde{V}}\int_{\tilde{V}}\mathrm{d}\mathbf{y}\left\langle \exp\left\{i\int_0^t \mathbf{k}(s)\cdot\mathbf{f}^*[\mathbf{Y}(s)]\mathrm{d}s\right\}\right\rangle_{\mathbf{Y}(0)=\mathbf{y}}\right)^m\\
&= \exp\left(-\bar{\rho}\tilde{V}+\bar{\rho}\int_{\tilde{V}}\mathrm{d}\mathbf{y}\left\langle \exp\left\{i\int_0^t \mathbf{k}(s)\cdot\mathbf{f}^*[\mathbf{Y}(s)]\mathrm{d}s\right\}\right\rangle_{\mathbf{Y}(0)=\mathbf{y}}\right)
\end{aligned}
\qquad \text{[S44]}
$$

using the independence, uniformity, and Poisson statistics of of the spatial Poisson process Φ. Upon taking the limit $\tilde{V}\to\infty$, Eq. [S44] becomes Eq. [5].

5. Formalism for forces depending on swimmer velocity

When $\mathbf{F}$ includes a velocity-dependence as in the hydrodynamic force Eq. [9], it is necessary to extend the microscopic density field to include velocity degrees of freedom

$$\rho(\mathbf{x},\mathbf{v},t)=\sum_{i=1}^{N}\delta(\mathbf{x}-\mathbf{Y}_i(t))\delta(\mathbf{v}-\dot{\mathbf{Y}}_i(t)), \qquad \text{[S45]}$$

which leads to the equation of motion of the tracer (cf. Eq. [1])

$$\dot{\mathbf{X}}(t)=\mu\int \mathrm{d}\mathbf{x}\int \mathrm{d}\mathbf{v}\,\mathbf{F}(\mathbf{x}-\mathbf{X}(t),\mathbf{v})\rho(\mathbf{x},\mathbf{v},t). \qquad \text{[S46]}$$

The further derivation follows the same steps as in the main text. The main ingredient is the n-point function to first order in $\bar{\rho}$, which now reads

$$\langle \rho(\mathbf{x}_1,\mathbf{v}_1,t_1)\cdots\rho(\mathbf{x}_n,\mathbf{v}_n,t_n)\rangle = \bar{\rho}\int \mathrm{d}\mathbf{y}\left\langle \delta(\mathbf{x}_1-\mathbf{Y}(t_1))\delta(\mathbf{v}_1-\dot{\mathbf{Y}}(t_1))\cdots\delta(\mathbf{x}_n-\mathbf{Y}(t_n))\delta(\mathbf{v}_n-\dot{\mathbf{Y}}(t_n))\right\rangle_{\mathbf{Y}(0)=\mathbf{y}}. \quad \text{[S47]}$$

Eq. [**S47**] can be shown following a similar calculation as in SI Appendix 2. The proof of Eq. [**S12**] is then unchanged, see SI Appendix 3. This can be easily seen in the heuristic proof, but also in the order-by-order calculation, since the additional $\dot{\mathbf{Y}}$-dependence in the force is simply contained in the explicit t-dependence in Eq. [**S23**]. The main result Eq. [**5**] is thus likewise valid.

For completeness, the solution of the two-body problem following from the Picard iteration of the ODE is here written out. The integrated form of the ODE is

$$\mathbf{X}(t) = \mu\int_0^t \mathrm{d}s\,\mathbf{F}(\mathbf{Y}(s)-\mathbf{X}(s),\dot{\mathbf{Y}}(s)), \quad \text{[S48]}$$

with $\mathbf{X}(0)=\mathbf{0}$. Up to second order we have then

$$\mathbf{X}^{*,2}(t) = \mu\int_0^t \mathrm{d}s\,\mathbf{F}\left(\mathbf{Y}(s)-\mu\int_0^s \mathrm{d}s'\,\mathbf{F}\left(\mathbf{Y}(s'),\dot{\mathbf{Y}}(s')\right),\dot{\mathbf{Y}}(s)\right) \quad \text{[S49]}$$

and thus

$$\mathbf{f}^*[\mathbf{Y}] \approx \mu\,\mathbf{F}\left(\mathbf{Y}(t)-\mu\int_0^t \mathrm{d}s'\,\mathbf{F}\left(\mathbf{Y}(s'),\dot{\mathbf{Y}}(s')\right),\dot{\mathbf{Y}}(s)\right). \quad \text{[S50]}$$

Taylor expansion in orders of μ then yields the first and second-order approximations

$$\mathbf{f}^*[\mathbf{Y}] \approx \mu\,\mathbf{F}(\mathbf{Y}(t),\dot{\mathbf{Y}}(t)) \quad \text{[S51]}$$

$$\mathbf{f}^*[\mathbf{Y}] \approx \mu\,\mathbf{F}(\mathbf{Y}(t),\dot{\mathbf{Y}}(t)) - \mu^2\nabla_{\mathbf{x}}\mathbf{F}(\mathbf{Y}(t),\dot{\mathbf{Y}}(t))^{\mathrm{T}}\int_0^t \mathrm{d}s\,\mathbf{F}(\mathbf{Y}(s),\dot{\mathbf{Y}}(s)). \quad \text{[S52]}$$

6. The special case of straight-line swimmer motion

In this section, I show how Eq. [**5**] recovers the literature results on the PDF of tracer displacements derived in (18) using an independent kick model and in (1) using a colored Poisson process. Both results rely on the specific assumption that swimmers move in straight lines instead of stochastically in time. Since the colored Poisson process of (1) also reproduces the displacement PDF of the static swimmer model of (19) for short times, this result is also recovered by Eq. [**5**].

The characteristic function of the displacements $\Delta\mathbf{X}_\tau = \mathbf{X}_\tau - \mathbf{X}_0$ is obtained by substituting $\mathbf{k}_u = \Theta(\tau-u)\mathbf{k}$ in Eq. [**5**], which yields

$$\ln\psi_{\Delta\mathbf{X}}(\mathbf{k}) = \bar{\rho}\int \mathrm{d}\mathbf{y}\left(\left\langle e^{i\int_0^t \mathrm{d}u\,\mathbf{k}\cdot\mathbf{f}^*[\mathbf{Y}_u]}\right\rangle_{\mathbf{Y}_0=\mathbf{y}} - 1\right). \quad \text{[S53]}$$

In this section, the trajectory of a single swimmer is given by

$$\mathbf{Y}(t) = \mathbf{y} + v_{\mathrm{A}}\hat{\mathbf{n}}t, \quad \text{[S54]}$$

where v_{A} is the constant swimming speed, $\mathbf{y}$ the initial position and $\hat{\mathbf{n}}$ the swimmer orientation (swimming direction), which remains unchanged in time. The initial position and orientation are sampled from uniform distributions.

A. Independent kick model. According to Eqs. [**S15**,**S16**] the quantity $\mathbf{f}^*$ in Eq. [**S53**] is now expressed as

$$\mathbf{f}^*[\mathbf{Y}_u] = \mu\mathbf{F}(\mathbf{y}+v_{\mathrm{A}}\hat{\mathbf{n}}u - \mathbf{X}^*(u)), \quad \text{[S55]}$$

where $\mathbf{X}^*(u)$ is the solution of the ODE

$$\dot{\mathbf{X}}(u) = \mu\mathbf{F}(\mathbf{y}+v_{\mathrm{A}}\hat{\mathbf{n}}u - \mathbf{X}(u)). \quad \text{[S56]}$$

Comparing with Eq. (1) in (18), we thus see that $\int_0^t \mathrm{d}u\,\mathbf{f}^*[\mathbf{Y}_u]$ corresponds to the "drift function" $\mathbf{\Delta}$ of a single tracer–swimmer scattering event and $\left\langle e^{i\int_0^t \mathrm{d}u\,\mathbf{k}\cdot\mathbf{f}^*[\mathbf{Y}_u]}\right\rangle_{\mathbf{Y}_0=\mathbf{y}}$ is the associated characteristic function conditional on a given initial position. The final result Eq. (24) in (18) is then identical to the inverse Fourier transform of $\psi_{\Delta\mathbf{X}}(\mathbf{k})$, Eq. [**S53**].

B. Colored Poisson process. In (1) a coloured Poisson process has been derived for the tracer dynamics, which specifies the characteristic functional Eq. [2] as

$$\ln \psi_{\dot{\mathbf{X}}}[\mathbf{k}] = \int_{-\infty}^{\infty} \mathrm{d}t' \int \mathrm{d}\mathbf{b} \int_{-\pi}^{\pi} \mathrm{d}\phi'\, \lambda(\mathbf{b}) \left(\exp\left\{ i \int_0^t \mathrm{d}s\, \mathbf{k}(s)\cdot \mathbf{f}^{\mathrm{O}}_{\mathbf{b},\phi'}(s-t') \right\} - 1 \right) \qquad \text{[S57]}$$

where $\mathbf{f}^{\mathrm{O}}_{\mathbf{b},\phi'}$ is the force shape function due to a single swimmer–tracer scattering event. The intensity of the Poisson process is given by $\lambda(\mathbf{b}) = \frac{\bar{\rho} v_{\mathrm{A}}}{4\pi b}$, which depends on the impact parameter vector $\mathbf{b}$. The force shape function $\mathbf{f}^{\mathrm{O}}_{\mathbf{b},\phi'}$ depends also on the injection angle ϕ' of the swimmer in the plane normal to $\mathbf{b}$ and has been derived in closed analytical form from the two-body interaction Eq. [S15] with the dipole force

$$\mathbf{F}_{\mathrm{hyd}}(\mathbf{x},\hat{\mathbf{n}}) = \frac{p}{\mathbf{x}^2}\left(3\frac{(\hat{\mathbf{n}}\cdot\mathbf{x})^2}{\mathbf{x}^2} - 1\right)\frac{\mathbf{x}}{|\mathbf{x}|}, \qquad \text{[S58]}$$

which is the leading-order hydrodynamic interaction force for force- and torque-free swimmers at low Reynolds numbers in the far-field regime (17). In Eq. [S58], the parameter p specifies the strength of the dipole force and $\hat{\mathbf{n}}$ the swimmer orientation $\hat{\mathbf{n}} = \dot{\mathbf{Y}}/|\dot{\mathbf{Y}}|$. The non-Markovian process Eq. [S57] reproduces the dynamical features of the tracer dynamics observed in simulations and experiments, predicting in particular a Lévy flight regime underlying the observed enhancement of the tracer diffusion (1). In the following I show that these results follow from Eq. [S53] for the special case of the swimmer dynamics given by the straight line motion Eq. [S54].

Starting with the first order approximation of $\mathbf{f}^*$, Eq. [S51], we obtain

$$\begin{aligned} \mathbf{f}^*[\mathbf{Y}(t)] &\approx \mu\, \mathbf{F}(\mathbf{Y}(t),\dot{\mathbf{Y}}(t)) \\ &= \mu\, \mathbf{F}_{\mathrm{hyd}}(\mathbf{y}+v_{\mathrm{A}}\hat{\mathbf{n}}t,\hat{\mathbf{n}}) \\ &= \frac{\mu\, p}{(\mathbf{y}+v_{\mathrm{A}}\hat{\mathbf{n}}t)^2}\left(3\frac{((\mathbf{y}+v_{\mathrm{A}}\hat{\mathbf{n}}t)\cdot\hat{\mathbf{n}})^2}{(\mathbf{y}+v_{\mathrm{A}}\hat{\mathbf{n}}t)^2} - 1\right)\frac{(\mathbf{y}+v_{\mathrm{A}}\hat{\mathbf{n}}t)}{\sqrt{(\mathbf{y}+v_{\mathrm{A}}\hat{\mathbf{n}}t)^2}} \end{aligned} \qquad \text{[S59]}$$

using Eqs. (S54,S58). Writing out the average in Eq. [S53] assuming an isotropic distribution of swimming directions $\hat{\mathbf{n}}$ yields

$$\ln \psi_{\dot{\mathbf{X}}}[\mathbf{k}] = \frac{\bar{\rho}}{4\pi}\int \mathrm{d}\mathbf{y} \oint \mathrm{d}\hat{\mathbf{n}} \left(\exp\left\{ i\mu \int_0^t \mathrm{d}s\, \mathbf{k}(s)\mathbf{F}_{\mathrm{hyd}}(\mathbf{y}+v_{\mathrm{A}}\hat{\mathbf{n}}t,\hat{\mathbf{n}}) \right\} - 1 \right) \qquad \text{[S60]}$$

The remaining integrals capture integrations over five degrees of freedom of the swimmer motion, which is the same dimensionality as for the integrals of Eq. [S57]. In order to implement a variable transformation between these sets of variables, I note that the impact parameter vector $\mathbf{b}$ of Eq. [S57] is constrained to be perpendicular to the swimmer direction $\hat{\mathbf{n}}$, such that there is only one degree of freedom (ϕ') describing the swimmer direction in addition to $\mathbf{b}$ (1). The vector $\mathbf{b}$ can thus be expressed as

$$\mathbf{b} = \mathbf{y} + v_{\mathrm{A}}\hat{\mathbf{n}}t_{\mathrm{c}}, \qquad t_{\mathrm{c}} = -\mathbf{y}\cdot\hat{\mathbf{n}}/v_{\mathrm{A}}, \qquad \text{[S61]}$$

where t_{c} is the time needed such that the swimmer starting at $\mathbf{y}$ satisfies $\mathbf{b}\cdot\hat{\mathbf{n}} = 0$. We obtain further $\mathbf{y}+v_{\mathrm{A}}\hat{\mathbf{n}}t = \mathbf{b}+v_{\mathrm{A}}\hat{\mathbf{n}}(t-t_{\mathrm{c}})$, which yields in Eq. [S60]

$$\mathbf{F}_{\mathrm{hyd}}(\mathbf{y}+v_{\mathrm{A}}\hat{\mathbf{n}}t,\hat{\mathbf{n}}) \quad = \quad \mathbf{F}_{\mathrm{hyd}}(\mathbf{b}+v_{\mathrm{A}}\hat{\mathbf{n}}(t-t_{\mathrm{c}}),\hat{\mathbf{n}}) \qquad \text{[S62]}$$

The term $\mathbf{F}_{\mathrm{hyd}}(\mathbf{b}+v_{\mathrm{A}}\hat{\mathbf{n}}t,\hat{\mathbf{n}})$ is already the first order approximation $f^{\mathrm{O}(1)}_{\mathbf{b},\phi'}(t)$ of the force shape function calculated in (1), which can be seen after expressing $\mathbf{b},\hat{\mathbf{n}}$ in spherical coordinates. To this end I introduce a spherical coordinate system $\mathbf{b} = (b\sin\theta\cos\phi, b\sin\theta\sin\phi, b\cos\theta)^{\mathrm{T}}$ with orthogonal unit vectors

$$\hat{\mathbf{e}}_b = \begin{pmatrix}\sin\theta\cos\phi\\ \sin\theta\sin\phi\\ \cos\theta\end{pmatrix}, \quad \hat{\mathbf{e}}_\theta = \begin{pmatrix}\cos\theta\cos\phi\\ \cos\theta\sin\phi\\ -\sin\theta\end{pmatrix}, \quad \hat{\mathbf{e}}_\phi = \begin{pmatrix}-\sin\phi\\ \cos\phi\\ 0\end{pmatrix}. \qquad \text{[S63]}$$

Expressing $\hat{\mathbf{n}}$ in terms of another set of spherical coordinates in this system via $\hat{\mathbf{n}} = \sin\theta'\cos\phi'\hat{\mathbf{e}}_\theta + \sin\theta'\sin\phi'\hat{\mathbf{e}}_\phi + \cos\theta'\hat{\mathbf{e}}_b$ (1), we see that the constraint $\mathbf{b}\cdot\hat{\mathbf{n}} = b\hat{\mathbf{e}}_b\cdot\hat{\mathbf{n}} = 0$ imposes $\cos\theta' = 0$ and thus $\sin\theta' = 1$ which yields

$$\hat{\mathbf{n}} = \begin{pmatrix}\cos\theta\cos\phi\cos\phi' - \sin\phi\sin\phi'\\ \cos\theta\sin\phi\cos\phi' + \cos\phi\sin\phi'\\ -\sin\theta\cos\phi'\end{pmatrix} \qquad \text{[S64]}$$

Noting that $(\mathbf{b}+v_{\mathrm{A}}\hat{\mathbf{n}}t)^2 = b^2 + v_{\mathrm{A}}^2 t^2$ we obtain

$$\mathbf{F}_{\mathrm{hyd}}(\mathbf{b}+v_{\mathrm{A}}\hat{\mathbf{n}}t,\hat{\mathbf{n}}) = \frac{p}{b^2+v_{\mathrm{A}}^2t^2}\left(3\frac{v_{\mathrm{A}}^2t^2}{b^2+v_{\mathrm{A}}^2t^2} - 1\right)\frac{(\mathbf{b}+v_{\mathrm{A}}\hat{\mathbf{n}}t)}{\sqrt{b^2+v_{\mathrm{A}}^2t^2}} \qquad \text{[S65]}$$

which together with Eq. [S64] is just Eq. (S69) in (1). The second order contribution $\mu^2\nabla_{\mathbf{x}}\mathbf{F}(\mathbf{Y},\dot{\mathbf{Y}})\int_0^t \mathrm{d}s\mathbf{F}(\mathbf{Y}(s),\dot{\mathbf{Y}}(s))$ of Eq. [S52] can now be calculated in the coordinates $(\mathbf{b},\phi')$ by integrating Eq. [S65] with respect to t, which yields a closed analytical result. This is equivalent to the method used in (1) to calculate the second order contribution to the force shape function $f^{\mathrm{O}(2)}_{\mathbf{b},\phi'}(t)$ and thus gives the same result.

The remaining step is to calculate the Jacobian of the transformation between the set of variables $(\mathbf{y},\hat{\mathbf{n}})$ used in Eq. [S60] and the variables $(t_{\mathrm{c}},\mathbf{b},\phi')$ used in Eq. [S57] (t_{c} is just the offset t', see Eq. [S62]). This is done easiest by keeping Cartesian coordinates for $(\mathbf{y},\hat{\mathbf{n}})$, whereby the orientational integral $\oint \mathrm{d}\hat{\mathbf{n}}$ can be expressed as a volume integral over a delta function enforcing the unit sphere surface

$$\begin{aligned}\frac{\bar{\rho}}{4\pi}\int \mathrm{d}\mathbf{y}\oint \mathrm{d}\hat{\mathbf{n}}\Big(...\Big) &= \frac{\bar{\rho}}{4\pi}\int \mathrm{d}\mathbf{y}\int \mathrm{d}n_x\int \mathrm{d}n_y\int \mathrm{d}n_z\delta\left(\sqrt{n_x^2+n_y^2+n_z^2}-1\right)\Big(...\Big)\\ &= \frac{\bar{\rho}}{4\pi}\int \mathrm{d}\mathbf{y}\int_{-1}^{1}\mathrm{d}n_x\int_{-\sqrt{1-n_x^2}}^{\sqrt{1-n_x^2}}\mathrm{d}n_y\frac{2}{\sqrt{1-n_x^2-n_y^2}}\Big(...\Big)\end{aligned} \qquad [S66]$$

The equations for the transformation are then (see Eqs. [S61,S64])

$$\mathbf{y} = \mathbf{b}-v_{\mathrm{A}}\hat{\mathbf{n}}t_{\mathrm{c}} \qquad [S67]$$

$$n_x = \cos\theta\cos\phi\cos\phi'-\sin\phi\sin\phi' \qquad [S68]$$

$$n_y = \cos\theta\sin\phi\cos\phi'+\cos\phi\sin\phi' \qquad [S69]$$

which gives the Jacobian determinant

$$|\mathcal{J}| = v_{\mathrm{A}}b\sin^2\theta\cos\phi'. \qquad [S70]$$

In addition, in the new coordinates

$$\sqrt{1-n_x^2-n_y^2} = \sin\theta\cos\phi'. \qquad [S71]$$

The remaining step is to find the correct boundaries in the new set of coordinates. To this end I note that $(\mathbf{y},\hat{\mathbf{n}})$ for a given swimmer is uniquely mapped onto a set $(t_{\mathrm{c}},\mathbf{b},\phi')$ with $t_{\mathrm{c}}\in[0,\infty)$ and $\phi'\in[-\pi,\pi]$. Overall, the transformation is thus

$$\begin{aligned}\frac{\bar{\rho}}{4\pi}\int \mathrm{d}\mathbf{y}\oint \mathrm{d}\hat{\mathbf{n}}\Big(...\Big) &= \int_0^\infty \mathrm{d}t_{\mathrm{c}}\int_0^\infty \mathrm{d}b\int_0^\pi \mathrm{d}\theta\int_{-\pi}^{\pi}\mathrm{d}\phi\int_{-\pi}^{\pi}\mathrm{d}\phi'\,\frac{\bar{\rho}v_{\mathrm{A}}}{2\pi}b\sin\theta\Big(...\Big)\\ &= \int_{-\infty}^{\infty}\mathrm{d}t_{\mathrm{c}}\int \mathrm{d}\mathbf{b}\int_{-\pi}^{\pi}\mathrm{d}\phi'\,\frac{\bar{\rho}v_{\mathrm{A}}}{4\pi\, b}\Big(...\Big),\end{aligned} \qquad [S72]$$

where the intensity $\lambda(\mathbf{b})$ of the coloured Poisson process is recovered as in Eq. [S57].

7. Universal time-independent statistics of the tracer velocity

The characteristic function of the tracer velocities $\dot{\mathbf{X}}(\tau)$ is obtained by substituting $\mathbf{k}_u=\delta(\tau-u)\mathbf{k}$ in Eq. [5]. In the adiabatic regime Eq. [7] we obtain

$$\ln\psi_{\dot{\mathbf{X}}}(\mathbf{k}) = \bar{\rho}\int \mathrm{d}\mathbf{y}\left(\left\langle e^{i\mu\mathbf{k}\cdot\mathbf{F}(\mathbf{Y}(\tau))}\right\rangle_{\mathbf{Y}_0=\mathbf{y}}-1\right). \qquad [S73]$$

Writing out the conditional average yields

$$\begin{aligned}\ln\psi_{\dot{\mathbf{X}}}(\mathbf{k}) &= \bar{\rho}\int \mathrm{d}\mathbf{y}\left(\int \mathrm{d}\mathbf{y}'G(\mathbf{y}',\tau|\mathbf{y})e^{i\mu\mathbf{k}\cdot\mathbf{F}(\mathbf{y}')}-1\right)\\ &= \bar{\rho}\int \mathrm{d}\mathbf{y}\int \mathrm{d}\mathbf{y}'\left(G(\mathbf{y}',\tau|\mathbf{y})e^{i\mu\mathbf{k}\cdot\mathbf{F}(\mathbf{y}')}-G(\mathbf{y}',\tau|\mathbf{y})\right)\\ &= \bar{\rho}\int \mathrm{d}\mathbf{y}'\left(e^{i\mu\mathbf{k}\cdot\mathbf{F}(\mathbf{y}')}-1\right)\end{aligned} \qquad [S74]$$

after introducing the marginal propagator of the swimmer position G, which satisfies translation invariance, i.e, $G(\mathbf{y}',\tau|\mathbf{y}) = \tilde{G}(\mathbf{y}'-\mathbf{y},\tau)$. The universal and time-independent result Eq. [S74] is due to the fact that the swimmer dynamics maintains the initially uniform distribution at all times leading to a time-independent distribution of the forces.

The validity of Eq. [S74] is tested in $2d$ numerical simulations of the tracer process given by the equation of motion [1] for the Coulomb-like interaction force

$$\mathbf{F}_{\mathrm{cb}}(\mathbf{x}) = \sigma\frac{\mathbf{x}}{|\mathbf{x}|^3}. \qquad [S75]$$

Such a radial force with a decay $\propto |\mathbf{x}|^{-2}$ has been shown to be a suitable model for a variety of effective interaction forces in active suspensions generated, e.g., due to chemical or phoretic interactions (20). Evaluating Eq. [**S74**] with Eq. [**S75**] for the tracer velocities projected on the x-axis (i.e., $\mathbf{k} = k\hat{\mathbf{e}}_x$) yields

$$\begin{aligned} \ln \psi_{\dot{\mathbf{X}}}(\mathbf{k}) &= \bar{\rho} \int_0^\infty \mathrm{d}r\, r \int_0^{2\pi} \mathrm{d}\alpha \left(e^{i\mu k \cos(\alpha)\sigma/r^2} - 1 \right). \\ &= -\pi\, \sigma\, \bar{\rho}\, \mu\, |k| \end{aligned} \quad [\mathrm{S76}]$$

and the PDF $P(\mathrm{v}_x)$ of the velocity projections $\mathrm{v_x} = \dot{\mathbf{X}}_\tau \cdot \hat{\mathbf{e}}_x$ shown in Fig. 1a) is given by the inverse Fourier transform

$$P(\mathrm{v}_x) = \frac{1}{2\pi} \int \mathrm{d}k\, e^{-ik\,\mathrm{v}_x - \pi\,\sigma\,\bar{\rho}\,\mu\,|k|}, \quad [\mathrm{S77}]$$

which is Eq. [**11**] with $c = \pi\sigma$.

In the simulations of Fig. 1, the following active particle models are considered as swimmer dynamics:

1. *Active Ornstein-Uhlenbeck particles (AOUPs):*

$$\dot{\mathbf{Y}}(t) = \mathbf{V}(t), \qquad \tau_{\mathrm{a}} \dot{\mathbf{V}}(t) = -\mathbf{V}(t) + \sqrt{D_v}\xi(t), \quad [\mathrm{S78}]$$

where $\xi(t)$ denotes Gaussian white noise with zero mean and covariance $\langle \xi(t)\xi(t')\rangle = \delta(t - t')$, τ_{a} the persistence time, and D_v the diffusion coefficient of the velocity.

2. *Active Brownian particles (ABPs):*

$$\dot{\mathbf{Y}}(t) = v_{\mathrm{A}} \hat{\mathbf{n}}(\theta), \qquad \dot{\theta}(t) = \sqrt{D_\theta}\xi(t), \quad [\mathrm{S79}]$$

where $\xi(t)$ is as before, D_θ the diffusion coefficient of the orientation angle, v_{A} the constant speed, and $\hat{\mathbf{n}} = (\cos(\theta), \sin(\theta))^{\mathrm{T}}$ the unit vector of the swimmer orientation.

3. *Run-and-tumble particles (RTPs):*

$$\dot{\mathbf{Y}}(t) = v_{\mathrm{A}} \hat{\mathbf{n}}(\theta), \quad [\mathrm{S80}]$$

with $\theta(t)$ given as a compound Poisson process with jump rate λ and angles chosen uniformly on $[0, 2\pi]$. The quantities v_{A} and $\hat{\mathbf{n}}$ are as before.

4. *Correlated angle particles (CAPs).* Here the angular coordinate undergoes an Ornstein-Uhlenbeck process:

$$\dot{\mathbf{Y}}(t) = v_{\mathrm{A}} \hat{\mathbf{n}}(\theta), \qquad \tau_{\mathrm{c}} \dot{\theta}(t) = -\theta(t) + \sqrt{D_\theta}\xi(t), \quad [\mathrm{S81}]$$

where τ_c is the persistence time of the angle and $\xi(t)$, D_θ, v_{A}, and $\hat{\mathbf{n}}$ are as before.

The simulation code is adapted from Ref. (1) and uses the same integrator, but replaces the straight line motion of the swimmers by a stochastic time evolution. The PDF of the projected velocities is sampled from $2 \cdot 10^5$ runs for each particle model and $\tau = 0.1, 1, 10, 20$ showing perfect agreement with Eq. [**S77**], see Fig. 1a) in the main text, as expected from the theory.

Further simulation details: initial positions are distributed uniformly in a rectangle of area L^2 with initial velocity distribution $p(\mathbf{v}) = \delta(v - v_{\mathrm{A}})\frac{1}{2\pi}$. The parameter values used are: time step 10^{-2}, side length $L = 800$, number of swimmers $N = 200$, mobility coefficient $\mu = 0.01$, Coulomb force strength $\sigma = 3$, (initial) swimming speed $v_{\mathrm{A}} = 1$, persistence time (AOUPs) $\tau_{\mathrm{a}} = 1$, velocity diffusion coefficient (AOUPs) $D_v = 1$, angular diffusion coefficient (ABPs, CAPs) $D_\theta = 1$, tumbling rate (RTPs) $\lambda = 2$, persistence time (CAPs) $\tau_{\mathrm{c}} = 0.1$. The PDF values obtained in the simulations are rescaled by a factor 10^4, i.e., Fig. 1 shows $P(\mathrm{v}_x) = \frac{1}{10^4}\tilde{P}\left(\frac{\tilde{v}_x}{10^4}\right)$, where $\tilde{P}, \tilde{v}_x$ are the simulation measurements. The same scaling is applied to the theoretical prediction Eq. [**S77**].

A. Forces depending on swimmer velocity. For forces in the form $\mathbf{F}(\mathbf{x}, \mathbf{v})$ that include a dependence on the swimmer velocity $\mathbf{v}$, Eq. [**S73**] becomes instead

$$\begin{aligned} \ln \psi_{\dot{\mathbf{X}}}(\mathbf{k}) &= \bar{\rho} \int \mathrm{d}\mathbf{y} \left(\left\langle e^{i\mu\mathbf{k}\cdot\mathbf{F}(\mathbf{Y}(\tau), \dot{\mathbf{Y}}(\tau))} \right\rangle_{\mathbf{Y}_0 = \mathbf{y}} - 1 \right) \\ &= \bar{\rho} \int \mathrm{d}\mathbf{y} \left(\int \mathrm{d}\mathbf{y}' \int \mathrm{d}\mathbf{v}' \int \mathrm{d}\mathbf{v}\, G(\mathbf{y}', \mathbf{v}', \tau|\mathbf{y}, \mathbf{v}) p(\mathbf{v}) e^{i\mu\mathbf{k}\cdot\mathbf{F}(\mathbf{y}', \mathbf{v}')} - 1 \right), \end{aligned} \quad [\mathrm{S82}]$$

where $p(\mathbf{v})$ is the initial distribution of velocities. Using again translation invariance of the propagator in the form $G(\mathbf{y}', \mathbf{v}', \tau|\mathbf{y}, \mathbf{v}) = \tilde{G}(\mathbf{y}' - \mathbf{y}, \mathbf{v}', \tau|\mathbf{v})$ yields

$$\ln \psi_{\dot{\mathbf{X}}}(\mathbf{k}) = \bar{\rho} \int \mathrm{d}\mathbf{y}' \left(\int \mathrm{d}\mathbf{v}' \int \mathrm{d}\mathbf{v}\, G_{\mathbf{v}}(\mathbf{v}', \tau|\mathbf{v}) p(\mathbf{v}) e^{i\mu\mathbf{k}\cdot\mathbf{F}(\mathbf{y}', \mathbf{v}')} - 1 \right), \quad [\mathrm{S83}]$$

introducing the marginal velocity propagator $G_{\mathbf{v}}(\mathbf{v}',\tau|\mathbf{v}) = \int \mathrm{d}\mathbf{y}' G(\mathbf{y}',\mathbf{v}',\tau|\mathbf{y},\mathbf{v})$.

Remarkably, Eq. [S83] becomes again time-independent under certain conditions. In order to continue, I focus on: (i) two dimensions; (ii) a force $\mathbf{F}(\mathbf{x},\hat{\mathbf{n}}(\theta))$ that depends on position $\mathbf{x}$ and the swimmer orientation $\hat{\mathbf{n}}$ described by a polar angle θ; and (iii) an initial velocity distribution with a uniform distribution of orientations $p(\mathbf{v}) = p(v)/(2\pi)$. This yields for Eq. [S83] in polar coordinates

$$\ln\psi_{\dot{\mathbf{X}}}(\mathbf{k}) \;=\; \bar{\rho}\int \mathrm{d}\mathbf{y}'\left(\int_0^\infty \mathrm{d}v' \int_0^\infty \mathrm{d}v \int_0^{2\pi}\mathrm{d}\theta' \int_0^{2\pi}\mathrm{d}\theta\, G_{\mathrm{p}}(v',\theta',\tau|v,\theta)p(v)\frac{1}{2\pi}e^{i\mu\mathbf{k}\cdot\mathbf{F}(\mathbf{y}',\hat{\mathbf{n}}(\theta'))} - 1\right). \quad \text{[S84]}$$

One can show that for ABPs, RTPs, and AOUPs the velocity propagator in polar coordinates $G_{\mathrm{p}}(v',\theta',\tau|v,\theta)$ is a function of the angular difference $\theta'-\theta$

$$G_{\mathrm{p}}(v',\theta',\tau|v,\theta) = \tilde{G}_{\mathrm{p}}(v',\theta'-\theta,\tau|v) \quad \text{[S85]}$$

and as a consequence we have

$$\int_0^\infty \mathrm{d}v' \int_0^{2\pi}\mathrm{d}\theta\, G_{\mathrm{p}}(v',\theta',\tau|v,\theta) \;=\; 1 \quad \text{[S86]}$$

due to normalization. Substituting Eq. [S86] into Eq. [S84] yields again a time-independent form of the characteristic function of tracer velocities

$$\ln\psi_{\dot{\mathbf{X}}}(\mathbf{k}) \;=\; \bar{\rho}\int \mathrm{d}\mathbf{y}' \int_0^{2\pi}\frac{\mathrm{d}\theta'}{2\pi}\left(e^{i\mu\mathbf{k}\cdot\mathbf{F}(\mathbf{y}',\hat{\mathbf{n}}(\theta'))} - 1\right). \quad \text{[S87]}$$

In the simulations of Fig. 1b) in the main text the interaction force is given as the hydrodynamic force $\mathbf{F}_{\mathrm{hyd}}$ of Eq. [S58]. Evaluating Eq. [S87] with Eq. [S58] for the tracer velocities projected on the x-axis (i.e., $\mathbf{k} = k\hat{\mathbf{e}}_x$) yields

$$\begin{aligned}\ln\psi_{\dot{\mathbf{X}}}(\mathbf{k}) &= \bar{\rho}\int_0^\infty \mathrm{d}y\, y \int_0^{2\pi}\mathrm{d}\alpha \int_0^{2\pi}\frac{\mathrm{d}\theta}{2\pi}\left(\exp\left\{-i\frac{\mu\, k\, p}{y^2}\cos(\alpha)\left(3\cos^2(\alpha-\theta)-1\right)\right\}-1\right).\\ &= -c_{\mathrm{h}}\,\bar{\rho}\,\mu\,|k\,p| \end{aligned} \quad \text{[S88]}$$

with the numerical value $c_{\mathrm{h}} \approx 3.17$. The PDF $P(\mathrm{v}_x)$ of the velocity projections $\mathrm{v}_{\mathrm{x}} = \dot{\mathbf{X}}_\tau\cdot\hat{\mathbf{e}}_x$ shown in Fig. [1]b) is then given by the inverse Fourier transform

$$P(\mathrm{v}_x) \;=\; \frac{1}{2\pi}\int \mathrm{d}k\, e^{-ik\,\mathrm{v}_x - c_{\mathrm{h}}\bar{\rho}\,\mu\,|k\,p|}, \quad \text{[S89]}$$

which is Eq. [11] with $c = c_{\mathrm{h}}|p|$. In the simulations of Fig. 1b) the dipole strength p in Eq. [S58] is set as $p = -1$.

The derivation shows that Eq. [S87] holds for a more restricted class of systems than Eq. [S74] and is in particular only valid in two dimensions and when the velocity propagator of the swimmer process satisfies Eq. [S85], which, nevertheless, captures a a wide range of processes.

As a counter-example, I consider the correlated angle particles of Eq. [S81], which do not satisfy Eq. [S85]. Comparing Eq. [S89] with the simulation data in Fig. 1b) confirms that Eq. [S87] holds for AOUPs, ABPs, and RTPs, but not for CAPs, as predicted by the theory. Of course, the theory is still valid for CAPs only the characteristic function is not given by the specific time-independent form Eq. [S87], but has to be evaluated from the general result Eq. [S83] using the associated velocity propagator.

The degree of violation of Eq. [S87] can be tuned by varying the persistence time of the CAPs for a given τ: for large persistence times, the swimmer process converges to that of the straight-line swimmer motion. In this case Eq. [S87] holds due to the fact that the dynamics trivially satisfies the underlying condition of a translation invariant angle Eq. [S85].

8. The Feynman-Kac equation determining $\tilde{Q}$

Functionals of the type of Eq. [14] have so far not been considered in the literature. In order to determine them from the solution of a Feynman-Kac (FK) type PDE, I first introduce the function

$$\tilde{Q}_t(\mathbf{y},\mathbf{v},\mathbf{a}|\mathbf{y}_0,\mathbf{v}_0;\mathbf{k}) \;=\; \left\langle \delta\left(\mathbf{a}-\mu\int_0^t \mathrm{d}s\,\mathbf{F}(\mathbf{Y}(s))\right) e^{-i\mu^2\int_0^t \mathrm{d}u\int_0^u \mathrm{d}s\,\mathbf{k}\cdot(\nabla\mathbf{F}(\mathbf{Y}_u)^{\mathrm{T}}\mathbf{F}(\mathbf{Y}_s))}\right\rangle_{\mathbf{Y}(0)=\mathbf{y}_0,\dot{\mathbf{Y}}(0)=\mathbf{v}_0}^{\mathbf{Y}(t)=\mathbf{y},\dot{\mathbf{Y}}(t)=\mathbf{v}}, \quad \text{[S90]}$$

where the brackets denote an average with respect to trajectories $\mathbf{Y}$ that start at position $\mathbf{y}_0$ with velocity $\mathbf{v}_0$ and end at time t at position $\mathbf{y}$ with velocity $\mathbf{v}$. Then it is clear that substituting $\tilde{Q}_t$ of Eq. [S90] into Eq. [15] will yield Eq. [14] by integration. In order to derive a PDE for $\tilde{Q}_t$ I first consider its Fourier transform

$$\begin{aligned}\hat{\tilde{Q}}_t(\mathbf{y},\mathbf{v},\mathbf{k}'|\mathbf{y}_0,\mathbf{v}_0;\mathbf{k}) &= \int \mathrm{d}\mathbf{a}\, e^{i\mathbf{k}'\cdot\mathbf{a}}\tilde{Q}_t(\mathbf{y},\mathbf{v},\mathbf{a}|\mathbf{y}_0,\mathbf{v}_0;\mathbf{k})\\ &= \left\langle e^{i\mu\int_0^t \mathrm{d}s\,\mathbf{k}'\cdot\mathbf{F}(\mathbf{Y}(s)) - i\mu^2\int_0^t \mathrm{d}u\int_0^u \mathrm{d}s\,\mathbf{k}\cdot(\nabla\mathbf{F}(\mathbf{Y}_u)^{\mathrm{T}}\mathbf{F}(\mathbf{Y}_s))}\right\rangle_{\mathbf{Y}(0)=\mathbf{y}_0,\dot{\mathbf{Y}}(0)=\mathbf{v}_0}^{\mathbf{Y}(t)=\mathbf{y},\dot{\mathbf{Y}}(t)=\mathbf{v}}\end{aligned} \quad \text{[S91]}$$

Considering the time interval $[0, t+\Delta t]$ with small Δt we obtain then

$$\begin{aligned}\hat{\tilde{Q}}_{t+\Delta t}(\mathbf{y},\mathbf{v},\mathbf{k}'|\mathbf{y}_0,\mathbf{v}_0;\mathbf{k}) &= \Big\langle e^{i\mu\int_t^{t+\Delta t}\mathrm{d}s\,\mathbf{k}'\cdot\mathbf{F}(\mathbf{Y}(s))-i\mu^2\int_t^{t+\Delta t}\mathrm{d}u\int_0^u\mathrm{d}s\,\mathbf{k}\cdot(\nabla\mathbf{F}(\mathbf{Y}_u)^{\mathrm{T}}\mathbf{F}(\mathbf{Y}_s))} \\ &\quad\times e^{i\mu\int_0^t\mathrm{d}s\,\mathbf{k}'\cdot\mathbf{F}(\mathbf{Y}(s))-i\mu^2\int_0^t\mathrm{d}u\int_0^u\mathrm{d}s\,\mathbf{k}\cdot(\nabla\mathbf{F}(\mathbf{Y}_u)^{\mathrm{T}}\mathbf{F}(\mathbf{Y}_s))}\Big\rangle_{\mathbf{Y}(0)=\mathbf{y}_0,\dot{\mathbf{Y}}(0)=\mathbf{v}_0}^{\mathbf{Y}(t+\Delta t)=\mathbf{y},\dot{\mathbf{Y}}(t+\Delta t)=\mathbf{v}} \\ &\approx \Big\langle\Big(1+i\mu\Delta t\,\mathbf{k}'\cdot\mathbf{F}(\mathbf{Y}_t)-i\mu^2\Delta t\int_0^t\mathrm{d}s\,\mathbf{k}\cdot(\nabla\mathbf{F}(\mathbf{Y}_t)^{\mathrm{T}}\mathbf{F}(\mathbf{Y}_s))\Big)e^{i\mu\int_0^t\mathrm{d}u\,\mathbf{k}'\cdot\mathbf{F}(\mathbf{Y}(u))} \\ &\quad\times e^{-i\mu^2\int_0^t\mathrm{d}u\int_0^u\mathrm{d}s\,\mathbf{k}\cdot(\nabla\mathbf{F}(\mathbf{Y}_u)^{\mathrm{T}}\mathbf{F}(\mathbf{Y}_s))}\Big\rangle_{\mathbf{Y}(0)=\mathbf{y}_0,\dot{\mathbf{Y}}(0)=\mathbf{v}_0}^{\mathbf{Y}(t+\Delta t)=\mathbf{y},\dot{\mathbf{Y}}(t+\Delta t)=\mathbf{v}}.\end{aligned} \quad [S92]$$

Since the expression inside the average now only depends on the swimmer dynamics up to time t, we can use the following decomposition property for Markov processes

$$\Big\langle\ldots\Big\rangle_{\mathbf{Y}(0)=\mathbf{y}_0,\dot{\mathbf{Y}}(0)=\mathbf{v}_0}^{\mathbf{Y}(t+\Delta t)=\mathbf{y},\dot{\mathbf{Y}}(t+\Delta t)=\mathbf{v}} = \int\mathrm{d}\mathbf{y}'\mathrm{d}\mathbf{v}'G(\mathbf{y},\mathbf{v},t+\Delta t|\mathbf{y}',\mathbf{v}',t)\Big\langle\ldots\Big\rangle_{\mathbf{Y}(0)=\mathbf{y}_0,\dot{\mathbf{Y}}(0)=\mathbf{v}_0}^{\mathbf{Y}(t)=\mathbf{y}',\dot{\mathbf{Y}}(t)=\mathbf{v}'} \quad [S93]$$

In Eq. [S93], I introduce the propagator G of the swimmer process $\mathbf{Y}$, which satisfies in general a Fokker-Planck equation of the type

$$\frac{\partial}{\partial t}G(\mathbf{y},\mathbf{v},t|\mathbf{y}_0,\mathbf{v}_0,0) = \mathcal{L}(\mathbf{y},\mathbf{v})G(\mathbf{y},\mathbf{v},t|\mathbf{y}_0,\mathbf{v}_0,0) \qquad G(\mathbf{y},\mathbf{v},0|\mathbf{y}_0,\mathbf{v}_0,0) = \delta(\mathbf{y}-\mathbf{y}_0)\delta(\mathbf{v}-\mathbf{v}_0). \quad [S94]$$

The detailed form of the operator $\mathcal{L}$ for ABPs, RTPs, AOUPs, and CAPs is given below. Using Eq. [S93] in Eq. [S92] yields

$$\begin{aligned}\hat{\tilde{Q}}_{t+\Delta t}(\mathbf{y},\mathbf{v},\mathbf{k}'|\mathbf{y}_0,\mathbf{v}_0;\mathbf{k}) &= \int\mathrm{d}\mathbf{y}'\mathrm{d}\mathbf{v}'G(\mathbf{y},\mathbf{v},t+\Delta t|\mathbf{y}',\mathbf{v}',t)\Big\langle\Big(1+i\mu\Delta t\,\mathbf{k}'\cdot\mathbf{F}(\mathbf{y}')-i\mu^2\Delta t\int_0^t\mathrm{d}s\,\mathbf{k}\cdot(\nabla\mathbf{F}(\mathbf{y}')^{\mathrm{T}}\mathbf{F}(\mathbf{Y}_s))\Big) \\ &\quad\times e^{i\mu\int_0^t\mathrm{d}u\,\mathbf{k}'\cdot\mathbf{F}(\mathbf{Y}(u))-i\mu^2\int_0^t\mathrm{d}u\int_0^u\mathrm{d}s\,\mathbf{k}\cdot(\nabla\mathbf{F}(\mathbf{Y}_u)^{\mathrm{T}}\mathbf{F}(\mathbf{Y}_s))}\Big\rangle_{\mathbf{Y}(0)=\mathbf{y}_0,\dot{\mathbf{Y}}(0)=\mathbf{v}_0}^{\mathbf{Y}(t)=\mathbf{y}',\dot{\mathbf{Y}}(t)=\mathbf{v}'}.\end{aligned} \quad [S95]$$

The inverse Fourier transform $\mathbf{k}'\to\mathbf{a}$ gives

$$\begin{aligned}\tilde{Q}_{t+\Delta t}(\mathbf{y},\mathbf{v},\mathbf{a}|\mathbf{y}_0,\mathbf{v}_0;\mathbf{k}) &= \int\mathrm{d}\mathbf{y}'\mathrm{d}\mathbf{v}'G(\mathbf{y},\mathbf{v},t+\Delta t|\mathbf{y}',\mathbf{v}',t)\Big\langle\Big(1-\mu\Delta t\,\mathbf{F}(\mathbf{y}')\cdot\nabla_{\mathbf{a}}-i\mu^2\Delta t\int_0^t\mathrm{d}s\,\mathbf{k}\cdot(\nabla\mathbf{F}(\mathbf{y}')^{\mathrm{T}}\mathbf{F}(\mathbf{Y}_s))\Big) \\ &\quad\times\delta\Big(\mathbf{a}-\mu\int_0^t\mathrm{d}s\,\mathbf{F}(\mathbf{Y}(s))\Big)e^{-i\mu^2\int_0^t\mathrm{d}u\int_0^u\mathrm{d}s\,\mathbf{k}\cdot(\nabla\mathbf{F}(\mathbf{Y}_u)^{\mathrm{T}}\mathbf{F}(\mathbf{Y}_s))}\Big\rangle_{\mathbf{Y}(0)=\mathbf{y}_0,\dot{\mathbf{Y}}(0)=\mathbf{v}_0}^{\mathbf{Y}(t)=\mathbf{y}',\dot{\mathbf{Y}}(t)=\mathbf{v}'} \\ &= \int\mathrm{d}\mathbf{y}'\mathrm{d}\mathbf{v}'G(\mathbf{y},\mathbf{v},t+\Delta t|\mathbf{y}',\mathbf{v}',t)\left(1-\mu\Delta t\,\mathbf{F}(\mathbf{y}')\cdot\nabla_{\mathbf{a}}-i\mu\Delta t\,\mathbf{k}\cdot\left(\nabla\mathbf{F}(\mathbf{y}')^{\mathrm{T}}\mathbf{a}\right)\right) \\ &\quad\times\tilde{Q}_t(\mathbf{y}',\mathbf{v}',\mathbf{a}|\mathbf{y}_0,\mathbf{v}_0;\mathbf{k})\end{aligned} \quad [S96]$$

In Eq. [S96] we further need to express the propagator G. A formal way of writing the solution of the Fokker-Planck equation Eq. [S94] is $G(\mathbf{y},\mathbf{v},t|\mathbf{y}_0,\mathbf{v}_0,0) = e^{t\mathcal{L}(\mathbf{y},\mathbf{v})}\delta(\mathbf{y}-\mathbf{y}_0)\delta(\mathbf{v}-\mathbf{v}_0)$, which, for small Δt becomes

$$G(\mathbf{y},\mathbf{v},\Delta t|\mathbf{y}_0,\mathbf{v}_0,0) = (1+\Delta t\mathcal{L}(\mathbf{y},\mathbf{v}))\delta(\mathbf{y}-\mathbf{y}_0)\delta(\mathbf{v}-\mathbf{v}_0). \quad [S97]$$

Thus substituting

$$G(\mathbf{y},\mathbf{v},t+\Delta t|\mathbf{y}',\mathbf{v}',t) = (1+\Delta t\mathcal{L}(\mathbf{y},\mathbf{v}))\delta(\mathbf{y}-\mathbf{y}')\delta(\mathbf{v}-\mathbf{v}') \quad [S98]$$

in Eq. [S96] and keeping only terms to first order Δt, yields the FK equation [16] in the continuum limit $\Delta t\to 0$.

For completeness, the explicit expressions of $\mathcal{L}$ for the active particle models simulated in Fig. 1 are given here, restricted to two dimensions.

1. For AOUPs we obtain from Eq. [S78]

$$\frac{\partial}{\partial t}G(\mathbf{y},\mathbf{v},t|\mathbf{y}_0,\mathbf{v}_0,0) = \left[-\mathbf{v}\cdot\nabla_{\mathbf{y}}+\frac{1}{\tau_{\mathrm{a}}}\nabla_{\mathbf{v}}\cdot\mathbf{v}+\frac{D_v}{2\tau_{\mathrm{a}}^2}\nabla_{\mathbf{v}}^2\right]G(\mathbf{y},\mathbf{v},t|\mathbf{y}_0,\mathbf{v}_0,0) \quad [S99]$$

2. For ABPs we obtain from Eq. [S79]

$$\frac{\partial}{\partial t}G(\mathbf{y},\theta,t|\mathbf{y}_0,\theta_0,0) = \left[-v_{\mathrm{A}}\hat{\mathbf{n}}(\theta)\cdot\nabla_{\mathbf{y}}+D_\theta\frac{\partial^2}{\partial\theta^2}\right]G(\mathbf{y},\theta,t|\mathbf{y}_0,\theta_0,0) \quad [S100]$$

3. For RTPs we obtain from Eq. [S80]

$$\frac{\partial}{\partial t}G(\mathbf{y},\theta,t|\mathbf{y}_0,\theta_0,0) = -v_\mathrm{A}\hat{\mathbf{n}}(\theta)\cdot\nabla_\mathbf{y}G(\mathbf{y},\theta,t|\mathbf{y}_0,\theta_0,0) + \frac{\omega}{2\pi}\int_0^{2\pi}\mathrm{d}\theta'\,G(\mathbf{y},\theta',t|\mathbf{y}_0,\theta_0,0) - \omega\,G(\mathbf{y},\theta,t|\mathbf{y}_0,\theta_0,0) \qquad \text{[S101]}$$

4. For CAPs we obtain from Eq. [S81]

$$\frac{\partial}{\partial t}G(\mathbf{y},\theta,t|\mathbf{y}_0,\theta_0,0) = \left[-v_\mathrm{A}\hat{\mathbf{n}}(\theta)\cdot\nabla_\mathbf{y} + \frac{1}{\tau_\mathrm{c}}\frac{\partial}{\partial\theta}\theta + \frac{D_\theta}{2\tau_\mathrm{c}^2}\frac{\partial^2}{\partial\theta^2}\right]G(\mathbf{y},\theta,t|\mathbf{y}_0,\theta_0,0) \qquad \text{[S102]}$$

A. Solution of the Feynman-Kac equation for straight-line swimmer motion. When the velocity of the swimmer does not fluctuate in time, the operator $\mathcal{L}$ that governs its time evolution is simply $\mathcal{L}(\mathbf{y},\mathbf{v}) = -\mathbf{v}\cdot\nabla_\mathbf{y}$. For a general force $\mathbf{F}(\mathbf{x},\mathbf{v})$, the FK equation [16] is then

$$\frac{\partial}{\partial t}\tilde{Q}_t = -\mathbf{v}\cdot\nabla_\mathbf{y}\tilde{Q}_t - \mu\mathbf{F}(\mathbf{y},\mathbf{v})\cdot\nabla_\mathbf{a}\tilde{Q}_t - i\mu\mathbf{k}\cdot\left(\nabla\mathbf{F}(\mathbf{y},\mathbf{v})^\mathrm{T}\mathbf{a}\right)\tilde{Q}_t, \qquad \text{[S103]}$$

with initial condition

$$\tilde{Q}_0(\mathbf{y},\mathbf{v},\mathbf{a}|\mathbf{y}_0,\mathbf{v}_0) = \delta(\mathbf{y}-\mathbf{y}_0)\delta(\mathbf{v}-\mathbf{v}_0)\delta(\mathbf{a}). \qquad \text{[S104]}$$

It is not strictly necessary to include the dependence on $\mathbf{v},\mathbf{v}_0$ here since the velocity does not change from its initial state, but I keep the formalism general. Eq. [S103] is a first-order PDE, which can be solved by the method of characteristics. In fact, one can directly see that the solution is given by

$$\begin{aligned}\tilde{Q}_t(\mathbf{y},\mathbf{v},\mathbf{a}|\mathbf{y}_0,\mathbf{v}_0;\mathbf{k}) &= \delta(\mathbf{v}-\mathbf{v}_0)\delta(\mathbf{y}-\mathbf{y}_0-\mathbf{v}t)\delta\left(\mathbf{a}-\mu\int_0^t\mathrm{d}s\,\mathbf{F}(\mathbf{y}_0+\mathbf{v}s,\mathbf{v})\right)\\ &\quad\times\exp\left\{-i\mu^2\int_0^t\mathrm{d}u\int_0^u\mathrm{d}s\,\mathbf{k}\cdot(\nabla\mathbf{F}(\mathbf{y}_0+\mathbf{v}u,\mathbf{v})^\mathrm{T}\mathbf{F}(\mathbf{y}_0+\mathbf{v}s,\mathbf{v}))\right\}\end{aligned} \qquad \text{[S105]}$$

With the integrations in Eq. [15] we obtain further

$$\begin{aligned}Q_t(\mathbf{y}_0;\mathbf{k}) &= \int\mathrm{d}\mathbf{y}\,\mathrm{d}\mathbf{v}\,\mathrm{d}\mathbf{v}_0\,\mathrm{d}\mathbf{a}\,p(\mathbf{v}_0)e^{i\mathbf{k}\cdot\mathbf{a}}\tilde{Q}_t(\mathbf{y},\mathbf{v},\mathbf{a}|\mathbf{y}_0,\mathbf{v}_0;\mathbf{k})\\ &= \int\mathrm{d}\mathbf{v}_0\,p(\mathbf{v}_0)\exp\left\{i\mu\int_0^t\mathrm{d}s\,\mathbf{F}(\mathbf{y}_0+\mathbf{v}_0s,\mathbf{v}_0)\right\}\\ &\quad\times\exp\left\{-i\mu^2\int_0^t\mathrm{d}u\int_0^u\mathrm{d}s\,\mathbf{k}\cdot(\nabla\mathbf{F}(\mathbf{y}_0+\mathbf{v}_0u,\mathbf{v}_0)^\mathrm{T}\mathbf{F}(\mathbf{y}_0+\mathbf{v}_0s,\mathbf{v}_0))\right\}\end{aligned} \qquad \text{[S106]}$$

For the hydrodynamic force Eq. [S58], Eq. [S106] recovers the colored Poisson process with the same force shape function as derived in (1). This can be seen by substituting Eq. [S106] in Eq. [5] and evaluating the remaining integrals following the discussion in SI Appendix 6.

B. Perturbation theory for swimmers moving as RTPs. When the swimmer process follows that of a RTP, the operator $\mathcal{L}$ is given as in Eq. [S101] and the FK equation [16] is then

$$\frac{\partial}{\partial t}\tilde{Q}_t = -\mathbf{v}\cdot\nabla_\mathbf{y}\tilde{Q}_t + \frac{\omega}{2\pi}\int_0^{2\pi}\mathrm{d}\theta\,\tilde{Q}_t - \omega\tilde{Q}_t - \mu\mathbf{F}(\mathbf{y},\mathbf{v})\cdot\nabla_\mathbf{a}\tilde{Q}_t - i\mu\mathbf{k}\cdot\left(\nabla\mathbf{F}(\mathbf{y},\mathbf{v})^\mathrm{T}\mathbf{a}\right)\tilde{Q}_t, \qquad \text{[S107]}$$

where the velocity is expressed in polar coordinates as $\mathbf{v} = v\hat{\mathbf{n}}(\theta)$ with $v = v_\mathrm{A}$ =const and the initial condition is

$$\tilde{Q}_0(\mathbf{y},\theta,\mathbf{a}|\mathbf{y}_0,\theta_0) = \delta(\mathbf{y}-\mathbf{y}_0)\delta(\theta-\theta_0)\delta(\mathbf{a}). \qquad \text{[S108]}$$

For simplicity I focus here on two dimensions. Introducing the linear first order differential operator $\mathcal{M}$ as

$$\mathcal{M} = -v\hat{\mathbf{n}}(\theta)\cdot\nabla_\mathbf{y} - \mu\mathbf{F}(\mathbf{y},v\hat{\mathbf{n}}(\theta))\cdot\nabla_\mathbf{a} - i\mu\mathbf{k}\cdot\left(\nabla\mathbf{F}(\mathbf{y},v\hat{\mathbf{n}}(\theta))^\mathrm{T}\mathbf{a}\right), \qquad \text{[S109]}$$

which is just the operator associated with the straight-line motion of the swimmers (compare Eq. [S109] with Eq. [S103]), Eq. [S107] can be written as

$$\frac{\partial}{\partial t}\tilde{Q}_t = \mathcal{M}\,\tilde{Q}_t + \frac{\omega}{2\pi}\int_0^{2\pi}\mathrm{d}\theta\,\tilde{Q}_t - \omega\tilde{Q}_t. \qquad \text{[S110]}$$

An RTP switches orientations with rate ω, which can be interpreted as perturbations of the straight line motion of the swimmers. This motivates the assumption that $\tilde{Q}_t$ can be expressed as a regular perturbation series in the form

$$\tilde{Q}_t = f_0 + \omega\, f_1 + \omega^2\, f_2 + ..., \tag{S111}$$

where the functions f_i are independent of ω. Substituting the series Eq. [S111] into Eq. [S110] yields the hierarchy of equations

$$\begin{aligned} \frac{\partial}{\partial t} f_0 &= \mathcal{M}\, f_0 && \text{[S112]}\\ \frac{\partial}{\partial t} f_1 &= \mathcal{M}\, f_1 + \frac{1}{2\pi}\int_0^{2\pi} \mathrm{d}\theta\, f_0 - f_0 && \text{[S113]}\\ \frac{\partial}{\partial t} f_2 &= \mathcal{M}\, f_2 + \frac{1}{2\pi}\int_0^{2\pi} \mathrm{d}\theta\, f_1 - f_1 && \text{[S114]}\\ &\vdots \end{aligned}$$

where now the integral terms are reduced to inhomogeneities in the PDEs. In terms of initial conditions it is sensible to assume that at $t=0$ the initial condition of $\tilde{Q}_t$, Eq. [S108], is satisfied by f_0 while all the other f_is vanish: $f_2 = f_3 = ... = 0$ for $t = 0$.

Since $\mathcal{M}$ is linear and of first order, closed form expressions of the f_i can be derived using the Duhamel principle as follows. Introducing the propagator $G(\mathbf{y},\theta,\mathbf{a},t|\mathbf{y}_0,\theta_0,\mathbf{a}_0,t_0)$ as solution of

$$\frac{\partial}{\partial t} G = \mathcal{M}\, G, \qquad G(\mathbf{y},\theta,\mathbf{a},t_0|\mathbf{y}_0,\theta_0,\mathbf{a}_0,t_0) = \delta(\mathbf{y}-\mathbf{y}_0)\delta(\theta-\theta_0)\delta(\mathbf{a}-\mathbf{a}_0) \tag{S115}$$

we find that the solution of the inhomogeneous equation

$$\frac{\partial}{\partial t}\tilde{G} = \mathcal{M}\,\tilde{G} + h, \qquad \tilde{G}(\mathbf{y},\theta,\mathbf{a},0) = 0 \tag{S116}$$

is given by

$$\tilde{G}(\mathbf{y},\theta,\mathbf{a},t) = \int \mathrm{d}\mathbf{y}' \int_0^{2\pi}\mathrm{d}\theta' \int \mathrm{d}\mathbf{a}' \int_0^t \mathrm{d}t'\, G(\mathbf{y},\theta,\mathbf{a},t|\mathbf{y}',\theta',\mathbf{a}',t') h(\mathbf{y}',\theta',\mathbf{a}',t'). \tag{S117}$$

To see this, one just has to calculate the derivative $\partial/\partial t$ of Eq. [S117] and use Eq. [S115], noting that $\mathcal{M}$ only acts on the $\mathbf{y},\mathbf{a}$ variables and can be taken outside of the integrals. Comparing Eqs. [S112]–[S117], we find the solutions:

$$f_0(\mathbf{y},\theta,\mathbf{a},t) = G(\mathbf{y},\theta,\mathbf{a},t|\mathbf{y}_0,\theta_0,0,0), \tag{S118}$$

which satisfies the initial condition Eq. [S108], and the other f_i are determined iteratively as

$$f_i(\mathbf{y},\theta,\mathbf{a},t) = \int \mathrm{d}\mathbf{y}' \int_0^{2\pi}\mathrm{d}\theta' \int \mathrm{d}\mathbf{a}' \int_0^t \mathrm{d}t'\, G(\mathbf{y},\theta,\mathbf{a},t|\mathbf{y}',\theta',\mathbf{a}',t') \left(\frac{1}{2\pi}\int_0^{2\pi}\mathrm{d}\theta'' f_{i-1}(\mathbf{y}',\theta'',\mathbf{a}',t') - f_{i-1}(\mathbf{y}',\theta',\mathbf{a}',t')\right) \tag{S119}$$

All that is left is thus to determine the propagator G by solving Eq. [S115], which is equivalent to Eq. [S103] with a slightly different initial condition. Using the method of characteristics, the solution for G is readily found (compare with Eq. [S105])

$$\begin{aligned} G(\mathbf{y},\theta,\mathbf{a}|\mathbf{y}_0,\theta_0) &= \delta(\theta-\theta_0)\delta(\mathbf{y}-\mathbf{y}_0-v\hat{\mathbf{n}}(\theta)t)\delta\left(\mathbf{a}-\mathbf{a}_0-\mu\int_0^t \mathrm{d}s\,\mathbf{F}(\mathbf{y}_0+v\hat{\mathbf{n}}(\theta)s, v\hat{\mathbf{n}}(\theta))\right)\\ &\times\exp\left\{-i\mu\int_0^t\mathrm{d}u\int_0^u\mathrm{d}s\,\mathbf{k}\cdot(\nabla\mathbf{F}(\mathbf{y}_0+v\hat{\mathbf{n}}(\theta)u, v\hat{\mathbf{n}}(\theta))^{\mathrm{T}}(\mathbf{a}_0+\mu\mathbf{F}(\mathbf{y}_0+v\hat{\mathbf{n}}(\theta)s, v\hat{\mathbf{n}}(\theta))))\right\} \end{aligned} \tag{S120}$$

which can also be verified by substitution into Eq. [S115].

9. Multiple passive tracer particles

Multiple passive tracer particles can be included in the framework by extending Eq. [1] as

$$\mathbf{X}_j(t) = -\mu\nabla U(\mathbf{X}_1,...,\mathbf{X}_n) + \mu\sum_{i=1}^N \mathbf{F}\left(\mathbf{Y}_i(t)-\mathbf{X}_j(t), \dot{\mathbf{Y}}_i(t)\right) \tag{S121}$$

for $j = 1,...,n$ tracers interacting via the potential function $U(\mathbf{X}_1,...,\mathbf{X}_n)$. The MSRJD formalism can then likewise be applied to the joint characteristic functional

$$\psi_{\{\dot{\mathbf{X}}_\mathbf{j}\}}[\mathbf{k}_1,...,\mathbf{k}_n] = \left\langle \exp\left\{ i\int_0^t \mathrm{d}u \sum_{j=1}^n \mathbf{k}_j(u)\cdot\dot{\mathbf{X}}_j(u)\right\}\right\rangle \tag{S122}$$

but the exact resummation of the perturbative series to first order in the swimmer density $\bar{\rho}$ becomes more challenging due to the additional interaction term. Approximations could be introduced by: (i) focusing on the simple case of straight-line swimmer motion; and (ii) assuming that the correlations between tracers induced by the swimmer field can be neglected.

Coming back to a single passive tracer, the characteristic functional Eq. [S57], derived from Eq. [5] for the straight-line swimmer approximation in SI Appendix 6, indicates that the tracer dynamics can be expressed as the colored Poisson process (in 3d) (1)

$$\dot{\mathbf{X}}(t) = \boldsymbol{\xi}(t) = \mu \sum_{i=1}^{N_t} \mathbf{f}^{\mathrm{O}}_{\mathbf{b},\phi'}(t - t_i), \tag{S123}$$

where N_t is a Poisson counting process with intensity $\lambda(\mathbf{b}) = \frac{\bar{\rho} v_{\mathrm{A}}}{4\pi\, b}$ and $\mathbf{f}^{\mathrm{O}}_{\mathbf{b},\phi'}$ is the force shape function due to a single swimmer–tracer scattering event for a swimmer described by the impact parameter vector $\mathbf{b}$ and injection angle ϕ' relative to the tracer, see SI Appendix 6.

A single tracer is thus described by a non-Gaussian colored noise-driven process with an (in general) non-exponential correlation decay. Under the assumptions (i,ii) above, Eq. [S121] can thus be approximated as

$$\dot{\mathbf{X}}_j(t) = -\mu \nabla U(\mathbf{X}_1, ..., \mathbf{X}_n) + \boldsymbol{\xi}_j(t), \tag{S124}$$

where the $\boldsymbol{\xi}_j$ are independent realizations of the noise Eq. [S123]. Interestingly, a system of interacting AOUPs follows likewise an equation of the form of Eq. [S124], where the $\boldsymbol{\xi}_j(t)$ are given as colored Gaussian noise. Such systems have been treated with the *unified colored noise approximation*, which yields an explicit formula for the non-equilibrium stationary PDF of the n-particle system (21, 22). A similar approach could be applied to determine the stationary PDF of Eq. [S124] using the extension of the unified colored noise approximation to colored Poisson noise (23). As a result, the theory could capture the long-range effective forces that arise in mixed suspensions of passive tracers and active swimmers (16, 24).

References

1. Kanazawa K, Sano TG, Cairoli A, Baule A (2020) Loopy Lévy flights enhance tracer diffusion in active suspensions. *Nature* 579:364–367.
2. Wu XL, Libchaber A (2000) Particle diffusion in a quasi-two-dimensional bacterial bath. *Phys. Rev. Lett.* 84:3017–3020.
3. Gachelin J, et al. (2013) Non-Newtonian viscosity of Escherichia coli suspensions. *Phys. Rev. Lett.* 110:268103.
4. Lagarde A, et al. (2020) Colloidal transport in bacteria suspensions: from bacteria collision to anomalous and enhanced diffusion. *Soft Matter* 16:7503–7512.
5. Kamdar S, et al. (2022) The colloidal nature of complex fluids enhances bacterial motility. *Nature* 603:819–823.
6. Petersen F, Hubbart JA (2020) Quantifying Escherichia coli and suspended particulate matter concentrations in a mixed-land use Appalachian watershed. *Water* 12:532.
7. Leptos KC, Guasto JS, Gollub JP, Pesci AI, Goldstein RE (2009) Dynamics of enhanced tracer diffusion in suspensions of swimming eukaryotic microorganisms. *Phys. Rev. Lett.* 103:198103.
8. Kurtuldu H, Guasto JS, Johnson KA, Gollub JP (2011) Enhancement of biomixing by swimming algal cells in two-dimensional films. *Proc. Natl. Acad. Sci. U.S.A.* 108:10391–10395.
9. Jeanneret R, Pushkin DO, Kantsler V, Polin M (2016) Entrainment dominates the interaction of microalgae with micron-sized objects. *Nat. Commun.* 7:12518.
10. Kurihara T, Aridome M, Ayade H, Zaid I, Mizuno D (2017) Non-Gaussian limit fluctuations in active swimmer suspensions. *Phys. Rev. E* 95:030601.
11. Wang X, In M, Blanc C, Nobili M, Stocco A (2015) Enhanced active motion of Janus colloids at the water surface. *Soft Matter* 11:7376–7384.
12. Sachs J, Kottapalli SN, Fischer P, Botin D, Palberg T (2021) Characterization of active matter in dense suspensions with heterodyne laser Doppler velocimetry. *Colloid Polym. Sci.* 299:269–280.
13. Singh K, Yadav A, Dwivedi P, Mangal R (2022) Interaction of active Janus colloids with tracers. *Langmuir* 38:2686–2698.
14. Granek O, Kafri Y, Tailleur J (2022) Anomalous transport of tracers in active baths. *Phys. Rev. Lett.* 129:038001.
15. Sainis SK, Merrill JW, Dufresne ER (2008) Electrostatic interactions of colloidal particles at vanishing ionic strength. *Langmuir* 24:13334–13337.
16. Angelani L, Maggi C, Bernardini ML, Rizzo A, Di Leonardo R (2011) Effective interactions between colloidal particles suspended in a bath of swimming cells. *Phys. Rev. Lett.* 107:138302.
17. Lauga E, Powers TR (2009) The hydrodynamics of swimming microorganisms. *Rep. Prog. Phys.* 72:096601.
18. Thiffeault JL (2015) Distribution of particle displacements due to swimming microorganisms. *Phys. Rev. E* 92:023023.
19. Zaid I, Mizuno D (2016) Analytical limit distributions from random power-law interactions. *Phys. Rev. Lett.* 117:030602.
20. Liebchen B, Mukhopadhyay AK (2021) Interactions in active colloids. *J. Phys. Condens. Matt.* 34:083002.
21. Maggi C, Marconi UMB, Gnan N, Di Leonardo R (2015) Multidimensional stationary probability distribution for interacting active particles. *Sci. Rep.* 5:10742.
22. Marconi UMB, Paoluzzi M, Maggi C (2016) Effective potential method for active particles. *Mol. Phys.* 114:2400–2410.
23. Chen L (1998) Systems driven by colored Poisson noise: unified colored noise approximation. *Commun. Theor. Phys.* 30:45.
24. Tanaka H, Lee AA, Brenner MP (2017) Hot particles attract in a cold bath. *Phys. Rev. Fluids* 2:043103.
